# Molecular characterization of emerging chicken and turkey parvovirus variants and novel strains in Guangxi, China

**DOI:** 10.1038/s41598-023-40349-5

**Published:** 2023-08-11

**Authors:** Yanfang Zhang, Bin Feng, Zhixun Xie, Minxiu Zhang, Qing Fan, Xianwen Deng, Zhiqin Xie, Meng Li, Tingting Zeng, Liji Xie, Sisi Luo, Jiaoling Huang, Sheng Wang

**Affiliations:** 1 Guangxi Key Laboratory of Veterinary Biotechnology, Guangxi Veterinary Research Institute, Nanning, 530000 Guangxi China; 2 Key Laboratory of China (Guangxi)-ASEAN Cross-Border Animal Disease Prevention and Control, Ministry of Agriculture and Rural Affairs of China, Nanning, 530000 Guangxi China

**Keywords:** Viral genetics, Genome

## Abstract

Avian parvoviruses cause several enteric poultry diseases that have been increasingly diagnosed in Guangxi, China, since 2014. In this study, the whole-genome sequences of 32 strains of chicken parvovirus (ChPV) and 3 strains of turkey parvovirus (TuPV) were obtained by traditional PCR techniques. Phylogenetic analyses of 3 genes and full genome sequences were carried out, and 35 of the Guangxi ChPV/TuPV field strains were genetically different from 17 classic ChPV/TuPV reference strains. The nucleotide sequence alignment between ChPVs/TuPVs from Guangxi and other countries revealed 85.2–99.9% similarity, and the amino acid sequences showed 87.8–100% identity. The phylogenetic tree of these sequences could be divided into 6 distinct ChPV/TuPV groups. More importantly, 3 novel ChPV/TuPV groups were identified for the first time. Recombination analysis with RDP 5.0 revealed 15 recombinants in 35 ChPV/TuPV isolates. These recombination events were further confirmed by Simplot 3.5.1 analysis. Phylogenetic analysis based on full genomes showed that Guangxi ChPV/TuPV strains did not cluster according to their geographic origin, and the identified Guangxi ChPV/TuPV strains differed from the reference strains. Overall, whole-genome characterizations of emerging Guangxi ChPV and TuPV field strains will provide more detailed insights into ChPV/TuPV mutations and recombination and their relationships with molecular epidemiological features.

## Introduction

According to the description of the International Committee on Viral Taxonomy (ICTV) 2021 (https://ictv.global/taxonomy), the eight genera of parvovirus under the traditional classification have recently been replaced by ten genera. *Aveparvovirus* is one of the genera and includes avian parvoviruses, such as chicken parvoviruses (ChPVs) and turkey parvoviruses (TuPVs)^1^. Parvoviruses are linear, single-stranded DNA viruses with a length of ~ 5 kb and at least 3 open reading frames (ORFs)^2^. The ORFs include a 5ʹ-ORF, a 3ʹ-ORF and a small ORF located between the other two. The 5ʹ-ORF encodes the nonstructural protein NS1, and the 3ʹ-ORF may encode the capsid proteins VP1, VP2 and VP3. Notably, the small ORF is a hypothetical protein (NP) and remains unknown^3^.

Parvoviruses were first identified in chickens via electron microscopy studies and by measurements of their genome sizes^4,5^. Trampel et al.^6^ reported the detection of TuPVs in turkeys and believed that TuPVs increased the incidence of intestinal diseases and bird mortality. The replication efficiency and error correction ability of carnivore parvovirus during the replication process were not strong, so the mutation rate of the virus genome is higher than that of general DNA virus genomes. Therefore, Shackelton^7^ believed that single-stranded DNA viruses undergo faster genetic evolution than double-stranded DNA viruses. Additionally, recombination events in parvoviruses have been detected, and these viruses have been found to show high genetic diversity^7,8^.

Avian parvoviruses are similar to parvoviruses in other vertebrates (e.g., cats, dogs, pigs, and cattle)^9–11^ and are often associated with gastrointestinal diseases, including runting–stunting syndrome (RSS) in chickens, poult enteritis and mortality syndrome (PEMS) in turkeys, Derzsy’s disease in young geese and beak atrophy and dwarfism syndrome (BADS) in different types of ducks^4,6,12–14^. Reports^4,15–17^ confirmed that ChPVs were associated with diarrhoea, suggesting that the viruses were important causative agents of intestinal diseases. It has been reported that the occurrence of cerebellar hypoplasia and viral enteritis in commercial chicken flocks is also associated with ChPVs^18,19^.

Recent ChPV and TuPV outbreaks began in the USA in 2008. Research by Zsak et al.^20^ suggested that ChPVs and TuPVs diverged from a common ancestor. A similar pattern of ChPV/TuPV infection was observed in chicken and turkey flocks from a Croatian (CRO) farm^21^. ChPV/TuPV infections were identified in intestinal samples from 15 chicken flocks and 2 turkey flocks sampled in Hungary between 2008 and 2010^16^. The prevalence of ChPV/TuPV was examined in individuals of commercial turkeys and flocks at different days of age in Poland from 2008 to 2011^22^, and the infection rates of TuPV and ChPV were found to be 29.4% and 22.2%, respectively. In South Korea, 34 commercial chicken flocks that experienced enteritis outbreaks were investigated for the presence of widespread enteroviruses between 2010 and 2012, and the ChPV positive rate was 26.5%^19^. Recent research by Nuñez et al.^23^ showed that ChPV was associated with diseases such as enteritis, pancreatitis and pancreatic atrophy. The ChPV and TuPV cases diagnosed in Guangxi, China, from 2014 to 2019 were the first indexed ChPV/TuPV infections in the southern region of China^24–26^ and caused enteric disorders and economic losses in the Guangxi poultry industry.

The complete coding regions of only a few classic ChPV and TuPV strains, such as the ChPV ABU-P1 strain and the TuPV 260 and TuPV 1078 strains^3^, as well as homologous strains, have been elucidated. The TuPV 260 and TuPV 1078 strains were originally isolated from turkeys with PEMS, and the ChPV ABU-P1 strain was originally isolated from chickens with RSS. As these three classic ChPV/TuPV strains continued to spread in poultry, they may have undergone natural selection and host adaptation to produce newly emerging ChPV/TuPV field strains or variants, as observed for other parvoviruses^27^.

Several intestinal disease-related pathogens have been confirmed as pathogens of RSS^16,28–34^. Nevertheless, the lack of a clear understanding of the complex aetiologies of RSS and PEMS and the existence of numerous virus types related to these syndromes are the main reasons why vaccines for RSS and PEMS have not been developed. Additional studies are needed to demonstrate the role of ChPVs in the aetiology of intestinal diseases. The current report aims to reveal the genetic diversity of ChPV and TuPV strains in China and to determine the phylogenetic relationships between these parvoviruses and highly similar strains to provide a reference for the prevention and treatment of RSS and PEMS.

## Results

### PCR confirmation of Guangxi ChPV and TuPV strains

The nonstructural (NS) and VP genes of the positive samples were amplified by PCR using primers targeting the conserved 561-bp NS1 region and 249-bp VP1/VP2 region, respectively. The epidemiological survey results are shown in Table 1. Table 1 shows that the total positive rate was 69.72%, while the positive rate of RSS-like cases was as high as 91.86%, and the positive rate of healthy chickens was 66.91%. The positive samples were further confirmed by sequencing the NS1 and VP genes. NCBI BLAST results showed that the samples had 98–100% homology with the ChPV ABU-P1 strain isolated from Hungary and the TuPV 260 strain isolated from the United States. The full genome sequence was successfully deduced from 32 PCR-positive chicken throat and cloacal swab samples and 3 PCR-positive turkey throat and cloacal swab samples using Sanger sequencing.

**Table 1:** Information on the samples and 35 ChPV/TuPV genome sequences.

Sampling area	No. flocks	No. swabs	Type	Positive rate		Strain name	Accession no	Age (days)	Collection date
				No. swabs (%)	No. RSS-like cases (%)				
Nanning	4	60	A	30 (50.00)	–	GX-CH-PV-1	KX084399	300	2014.10.10
Nanning	2	60	B	23 (38.33)	–	GX-CH-PV-2	KX084400	152	2014.10.10
Nanning	7	84	B	40 (47.62)	4/5 (80.00)	GX-CH-PV-4	KX084401	53	2014.10.31
Nanning	3	48	B	47 (97.92)	–	GX-CH-PV-5	KX133426	19	2014.12.30
Nanning	7	84	C	74 (88.10)	18/20(90.00)	GX-CH-PV-6	KX133427	62	2014.12.30
Wuzhou	5	60	B	41 (68.33)	–	GX-CH-PV-7	KU523900	20	2015.03.20
Yulin	5	60	C	40 (66.67)	10/12 (83.33)	GX-CH-PV-8	KX133415	75	2015.07.24
Yulin	6	90	C	80 (88.89)	14/16 (87.50)	GX-CH-PV-9	KX133416	72	2015.07.24
Wuzhou	4	48	C	39 (54.17)	8/8 (100.00)	GX-CH-PV-10	KX133417	57	2015.07.24
Wuzhou	4	48	B	32 (66.67)	–	GX-CH-PV-11	KX133418	43	2015.08.12
Nanning	2	48	C	46 (95.83)	10/10 (100.00)	GX-CH-PV-12	KX133419	20	2015.08.12
Qinzhou	4	48	B	30 (62.50)	–	GX-CH-PV-13	KX133420	140	2015.08.13
Qinzhou	4	48	B	25 (52.08)	–	GX-CH-PV-14	KX133421	24	2015.08.13
Nanning	4	48	E	41 (85.42)	11/12 (91.67)	GX-CH-PV-15	KX133422	29	2015.10.09
Nanning	2	24	E	12 (50.00)	–	GX-CH-PV-16	KX133423	266	2015.10.09
Nanning	4	48	E	47 (97.92)	9/10 (90.00)	GX-CH-PV-17	KX133424	21	2015.10.10
Liuzhou	4	48	E	48 (100.00)	11/12 (91.67)	GX-CH-PV-18	KX133425	22	2015.10.10
Beihai	3	24	C	16 (66.67)	–	GX-CH-PV-19	MG602509	120	2016.09.30
Baihai	3	24	C	16 (66.67)	–	GX-CH-PV-20	MG602510	130	2016.09.30
Guilin	3	24	C	14 (58.33)	4/4 (100.00)	GX-CH-PV-21	MG602511	26	2016.09.30
Guilin	3	24	C	14 (58.33)	4/4 (100.00)	GX-CH-PV-22	MG602512	16	2016.11.11
Fangchenggang	4	48	C	33 (68.75)	–	GX-CH-PV-23	MG602513	120	2016.11.11
Fangchenggang	4	40	C	22 (55.00)	9/10 (90.00)	GX-CH-PV-24	MG602514	110	2017.04.21
Fangchenggang	2	20	C	10 (50.00)	2/2 (100.00)	GX-CH-PV-25	MG602515	100	2017.04.21
Nanning	2	20	C	12 (60.00)	5/5 (100.00)	GX-CH-PV-26	MG602516	77	2017.07.18
Nanning	4	48	B	4 (8.33)	–	GX-CH-PV-27	MG602517	280	2017.07.18
Nanning	4	48	C	40 (83.33)	10/12 (83.33)	GX-CH-PV-28	MG602518	80	2017.07.04
Qinzhou	4	48	C	26 (54.17)	8/8 (100.00)	GX-CH-PV-29	MG602519	40	2017.08.03
Qinzhou	4	48	C	39 (81.25)	10/10 (100.00)	GX-CH-PV-30	MG602520	20	2017.08.03
Nanning	1	12	D	2 (16.67)	–	GX-Tu-PV-1	KX084396	20	2015.03.10
Nanning	2	24	D	24 (100.00)	11/12 (91.67)	GX-Tu-PV-2	KX084397	90	2015.04.16
Nanning	3	48	D	46 (95.83)	–	GX-Tu-PV-3	KX084398	150	2015.04.16
Nanning	3	24	B	17 (70.83)	–	GX-CH-PV-31	OQ437199	120	2021.10.11
Nanning	3	24	B	18 (75.00)	–	GX-CH-PV-32	OQ437200	125	2022.05.16
Nanning	3	24	B	16 (66.67)	–	GX-CH-PV-33	OQ437201	130	2022.09.22
Total	126	1526		1064 (69.72)	158/172 (91.86)				

A: Layer chicken; B: Breeder chicken; C: Broiler chicken; D: Broiler turkey; E: Exotic broiler chicken; Exotic chickens = A + E; Native chickens = B + C.

Each chicken flock had between 8,000 and 12,000 chickens; each turkey flock had between 600 and 1000 turkeys.

### Overall features of the genomes

The genomes of the Guangxi ChPV and TuPV strains ranged from 4612 to 4642 bp in length. The approximate GC content of the genomes was 42.88%, and they each contained 3 segments encoding 4 viral proteins. The genomic segments ranged from 305 bp (NP1) to 2085 bp (NS1) in length, and ORF analysis of the nucleotide (nt) sequences indicated that 2 of the 3 genome segments encoded a single ORF, which were all similar to those of the ChPV/TuPV reference strains. The first ORF was predicted to encode 2 putative proteins (NS1 on NS1 and NP1 on NP1) ranging in size from 101 to 695 amino acids (aa). The 2028-bp VP segment was found to contain two partially overlapping genes encoding VP1 (2028 bp, 676 aa) and VP2 (1611 bp, 537 aa).

Comparisons of the similarities between the nt sequences of the Guangxi ChPV/TuPV strains and those of 17 ChPV/TuPV reference strains revealed that all 3 segments identified in the Guangxi ChPV/TuPV strains showed varying degrees of homology with the reference ChPV/TuPV strains. The 35 Guangxi isolates showed 79.4–99.7% nt identity with each other, and 78.7–99.7% nt identity with 11 classic ChPV reference strains, including the ChPV ABU-P1, ChPV ADL120686, ChPV ADL120019, ChPV ADL120035, ChPV 367, ChPV 736, ChPV 798, ChPV 841, ChPV ParvoD62/2013, ChPV ParvoD11/2007, and ChPV IPV strains, and 6 classic TuPV reference strains, including the TuPV 260, TuPV 1078, TuPV 1030, TuPV 1085, TuPV 1090 and TuPV JO11 strains.

### Nucleotide and amino acid comparisons

Comparing the nt and aa sequences of the NS1 gene revealed high sequence identities between the 35 Guangxi ChPV and TuPV strains and 11 ChPV reference strains and 6 TuPV reference strains. GPV (accession no. NC_001701 from the USA) and DPV (accession no. U22967 from Hungary) were used as outgroups. The accession numbers of the reference sequences of ChPVs/TuPVs are listed in Supplementary Tables S1 and S2. Homology analysis of the NS1 gene showed that the homologies of the nt and deduced aa sequences of the 35 Guangxi isolates were 88.1–99.9% and 89.1–100.0%, respectively. The nt sequence alignment between the ChPV and TuPV strains from Guangxi and those from other countries revealed 85.2–99.9% similarity, and the aa sequences showed 87.8–100% identities. The sequence identity of the NP1-encoding genes was the highest (> 95%); however, the role of this putative protein remains unknown^3^.

Compared with the genome fragments encoded by NS1 and NP1, the VP-encoding segments showed higher genetic diversity. For the VP1 protein, the Guangxi ChPV and TuPV strains showed similar identities with the ChPV/TuPV reference strains (nt, 73–98%; aa, 77.1–100%). Conversely, the VP2 protein shared the lowest identity with the ChPV 367 strain and the highest identity with the TuPV JO11 strain (nt, 72–98%; aa, 76.9–100%). For the VP1 gene, the homologies of the nt and deduced aa sequences of the 32 Guangxi isolates were 72.6–99.9% and 78.0–99.7%, respectively, and the homologies between the ChPV and TuPV isolates from Guangxi and those from other countries were 72.7–99.9% and 77.2–99.6%, respectively. For the VP2 gene, the homologies of the nt and deduced aa sequences of the 32 Guangxi isolates were 70.7–99.9% and 77.3–99.6%, respectively, and the homologies between the Guangxi ChPV and TuPV isolates and those from other countries were 71.1–99.9% and 77.1–99.4%, respectively.

### Sequence analysis

Interestingly, an 8-nt (TTATTTTG) deletion (corresponding to nts 2778 to 2785 in the NP1 gene of strain ABU-P1) was observed in all of the ChPV and TuPV strains except for the GX-CH-PV-1 and ChPV 841 strains and the TuPV 1078, 1085, 1090, GX-Tu-PV-1, GX-Tu-PV-2, and GX-Tu-PV-3 strains (see Supplementary Fig. S1). Additionally, a 4-nt (CTAA) deletion (corresponding to nt 2789 to 2792 in the NP1 gene of strain ABU-P1) was found in all of the ChPV and TuPV strains except for the GX-CH-PV-1 and ChPV 841 strains and the TuPV 1078, 1085, 1090, GX-Tu-PV-1, GX-Tu-PV-2, and GX-Tu-PV-3 strains. Moreover, a 9-nt (TCCATAATG) deletion (corresponding to nt 3275 to 3283 in the VP1 gene of strain ABU-P1) was found in all of the ChPV and TuPV strains except for the GX-CH-PV-1 and ChPV 841 strains and the TuPV 1078, 1085, 1090, GX-Tu-PV-1, GX-Tu-PV-2, and GX-Tu-PV-3 strains (see Supplementary Fig. S2). Finally, a 3-nt (GAA) deletion (corresponding to nt 3570 to 3572 in the VP2 gene of strain ABU-P1) was observed in all of the ChPV and TuPV strains except for the GX-CH-PV-1 and ChPV 841 strains and the TuPV 1078, 1085, 1090, GX-Tu-PV-1, GX-Tu-PV-2, and GX-Tu-PV-3 strains (see Supplementary Fig. S3).

Sixteen of the Guangxi ChPV/TuPV strains (i.e., GX-CH-PV-4, GX-CH-PV-6, GX-CH-PV-7, GX-CH-PV-13, GX-CH-PV-14, GX-CH-PV-15, GX-CH-PV-17, GX-CH-PV-18, GX-CH-PV-20, GX-CH-PV-21, GX-CH-PV-22, GX-CH-PV-25, GX-CH-PV-27, GX-CH-PV-28, GX-CH-PV-29 and GX-CH-PV-30) sequenced in this study all contained the putative VP3 start codon (spanning nts 3919 to 3921 in the ABU-P1 strain), which has also been identified in the ChPV ABU-P1, ChPV 367, ChPV 736, ChPV 798 and TuPV 260 strains (see Supplementary Fig. S4). Therefore, the VP3 protein of ChPV is not produced by alternative splicing of ORF2.

A highly conserved phosphate-binding loop (P-loop) motif (aa 392 to 399, GPANTGKT) and NTP binding motif (aa 436 to 437, EE) corresponding to the NS1 gene of strain ABU-P1 were present in all Guangxi ChPV/TuPV strains. The start codons for VP1 (nts 2998 to 3000; ABU-P1) and VP2 (nts 3415 to 3417; ABU-P1), the leucine residue (L; aa 293, VP1 of ABU-P1; aa 152, VP2 of ABU-P1), and the region of fivefold cylinders (LQVIQKTVTDSGTQYSND; aa 275 to 292, VP1 of ABU-P1; aa 134 to 151, VP2 of ABU-P1) were identified (see Supplementary Fig. S5), but a glycine-rich sequence (GGGGGGGGG; aa 164 to 172, VP1 of ABU-P1; aa 23 to 31, VP2 of ABU-P1) was identified in GX-TU-PV-1, GX-TU-PV-2, GX-TU-PV-3, GX-CH-PV-1 and GX-CH-PV-24, while TVGGGGGGG was identified in other Guangxi ChPV isolates.

### Phylogenetic analysis

**Figure 1:**
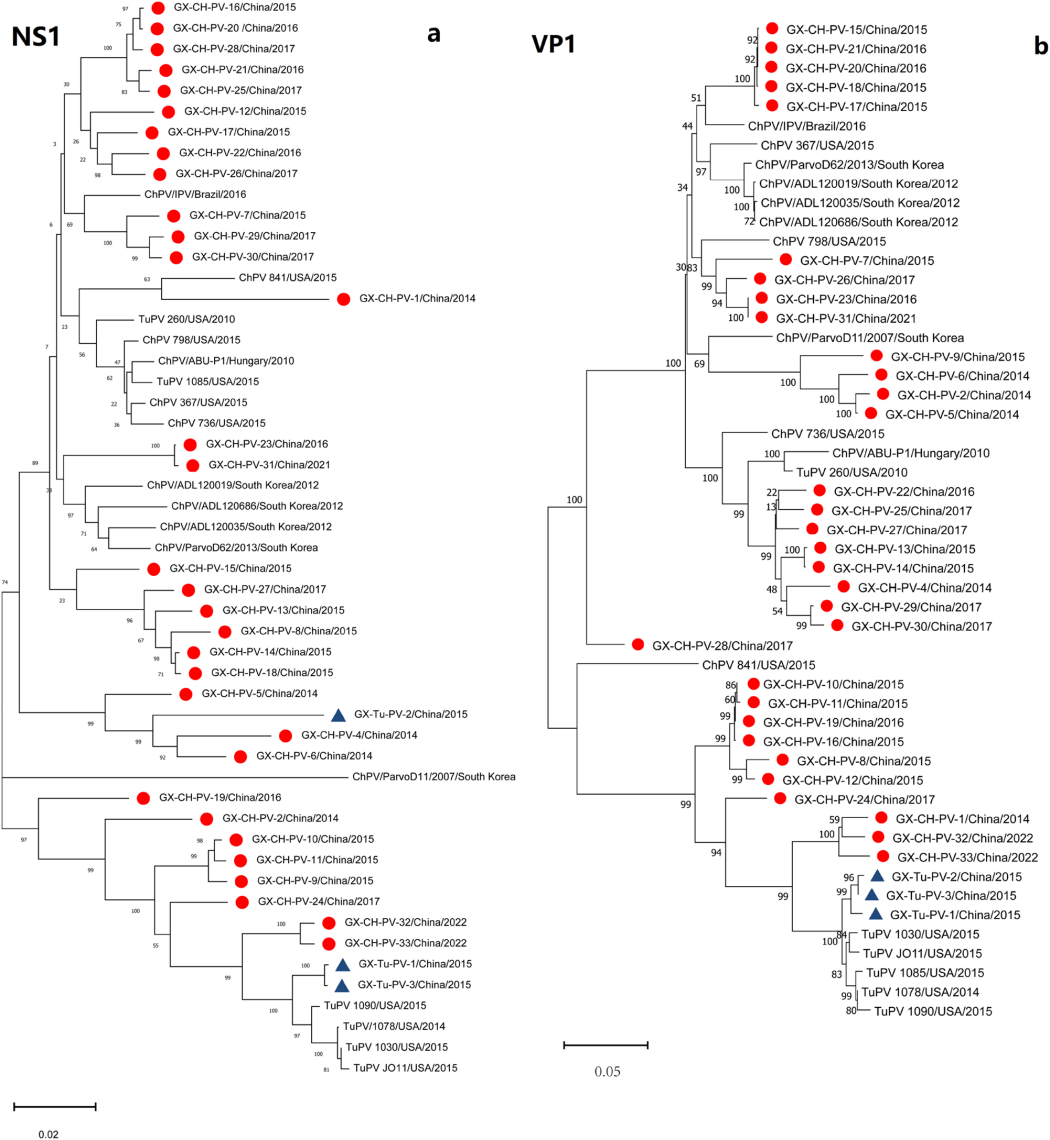

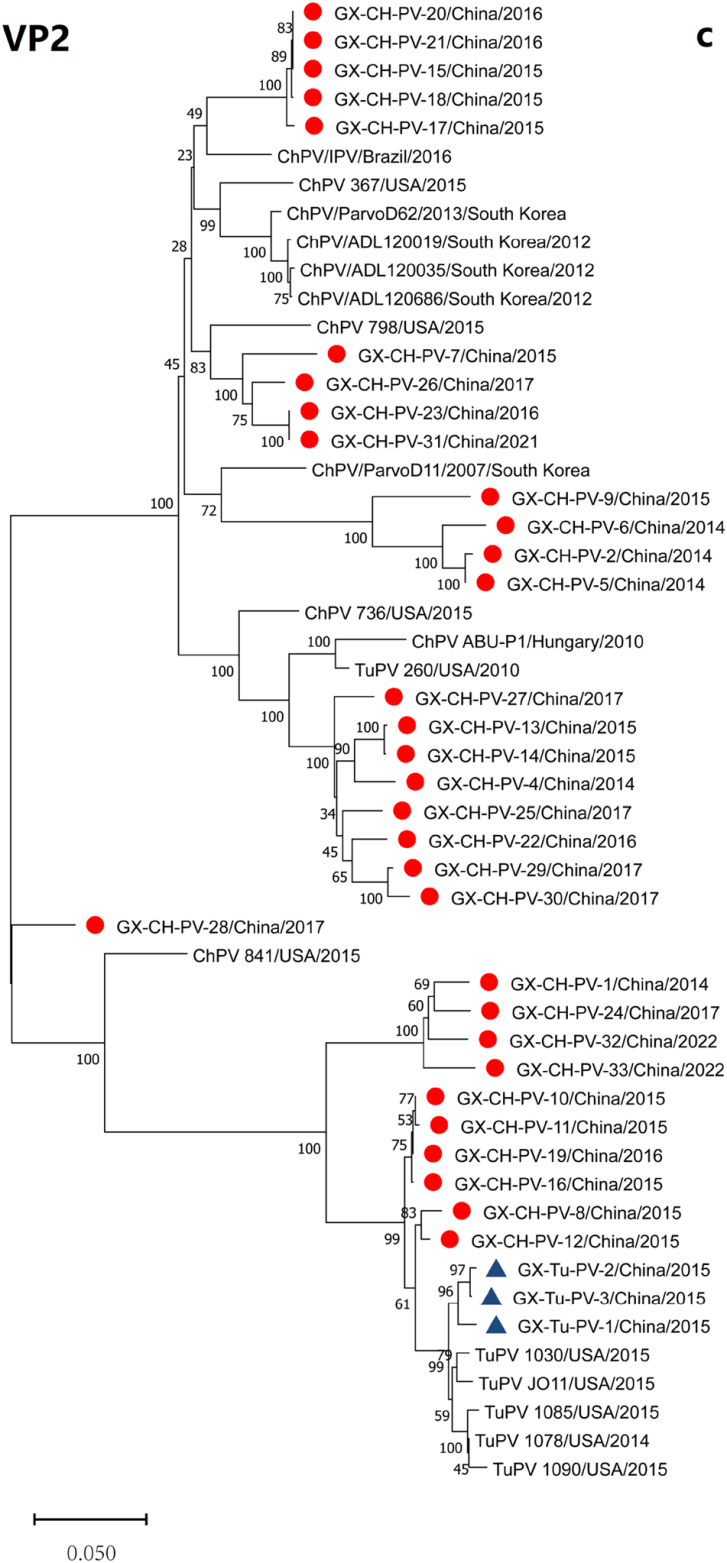

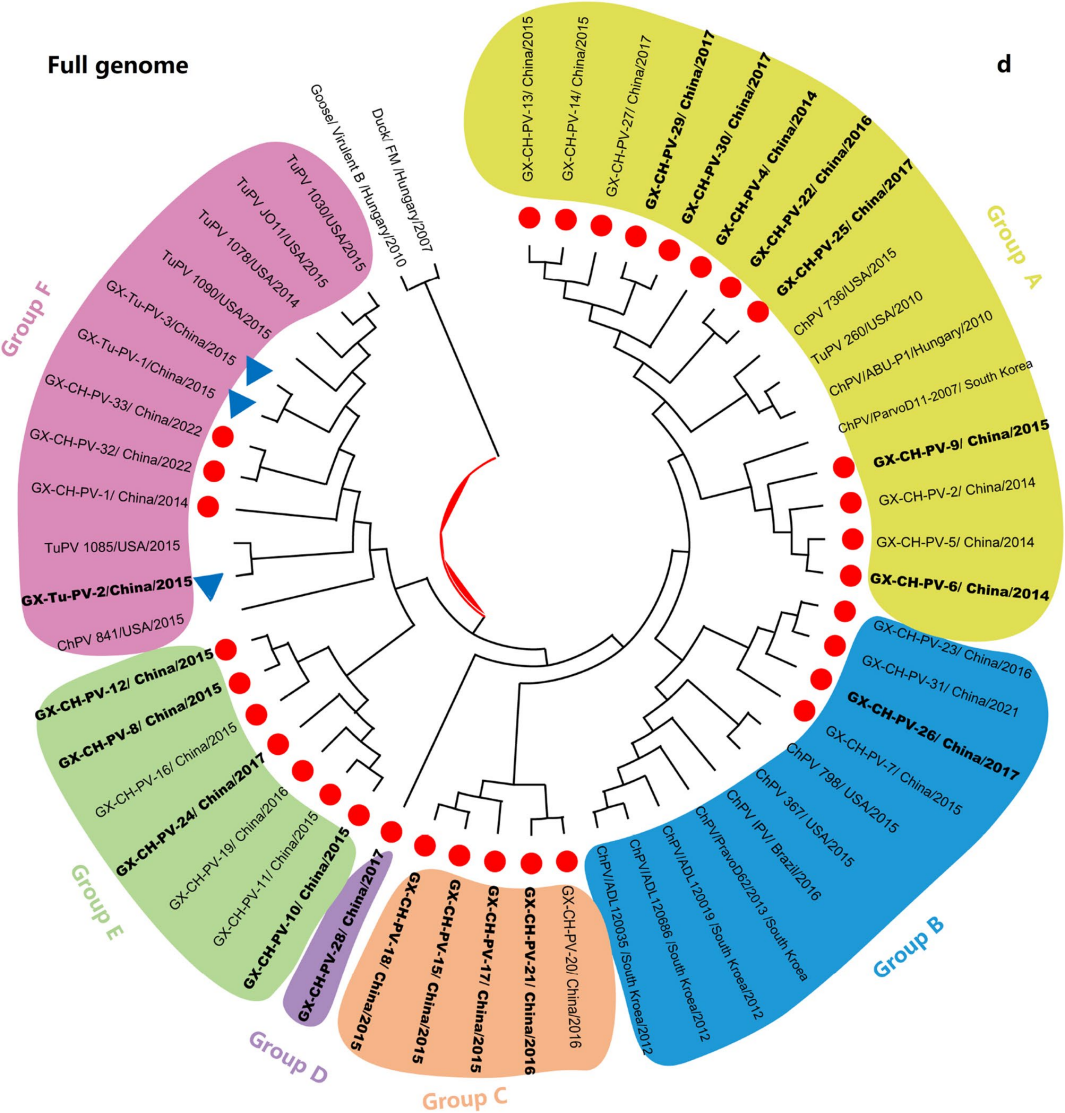
Phylogenetic trees constructed using the nucleotide sequences of 3 homologous genome segments (NS1, VP1 and VP2) (**a**–**c**) and the full genomes (**d**) of ChPV and TuPV, with DPV (GenBank accession number: U22967) and GPV (GenBank accession number: NC_001701) as outgroups. To construct the trees, 1000 bootstrap replicates were used. The bar indicates the genetic distance between sequences, and bootstrap values are shown at the nodes. Red filled circle and blue filled triangle represent the Guangxi ChPV strains and TuPV strains, respectively. The ChPVs/TuPVs in bold black font indicate chickens with RSS-like symptoms (**d**).

Evolutionary relationships between the Guangxi ChPV and TuPV strains and different members of the *Aveparvovirus* genus, including DPV and GPV, which were used as outgroup controls, were determined by phylogenetic analysis. Based on the nt sequences of the NS1, VP1 and VP2 genome segments and the whole ChPV/TuPV genomes, the neighbour-joining method with 1000 bootstrap replicates was used to construct the phylogenetic trees (Fig. 1a–d). All the constructed phylogenetic trees showed marked divergence between the Guangxi ChPV and TuPV strains and the other reference ChPV and TuPV strains. For the 3 genome segments, the vast majority of the ChPV strains formed a host-associated group (except for strains TuPV 260, ChPV 841, GX-Tu-PV-2, and GX-CH-PV-1) that differed from the turkey strains, the FM duck strain, and the virulent B goose strain. Furthermore, the segments encoding the VP1/VP2 proteins exhibited noticeably higher divergence than NS1 in the ChPV and TuPV strains, as indicated by sequence comparisons. A phylogenetic tree based on the VP gene revealed that the 35 Guangxi ChPV and TuPV isolates sequenced in our research clustered into 5 ChPV/TuPV groups designated Groups A, B, C, D, E and F (Fig. 1d). Genotyping cluster A, which consisted of 12 Guangxi ChPV field strains, included 2 prototype ChPV and TuPV strains (strains ABU-P1 and 260), 1 *Gallus gallus* enteric parvovirus isolate (strain 736) from the USA and 1 prototype ChPV strain (strains ParvoD11-2007) from South Korea; genotyping cluster B, which consisted of 4 Guangxi ChPV field strains, included 7 prototype ChPV strains from the USA, South Korea and Brazil; and genotyping cluster F, which consisted of 3 Guangxi TuPV and 3 ChPV field strains, included 6 prototype ChPV and TuPV strains all from the USA. Eighteen of the 35 ChPV/TuPV isolates were identified as Group A and Group F, while 3 Guangxi ChPV and 3 Guangxi TuPV field strains of Group F were field variants and were distinct from the prototype ChPV (strain 841) and TuPV (strains 1085, 1078, 1090, 1030 and JO11) strains, all from the USA. More importantly, 3 novel ChPV/TuPV groups (Groups C, D and E) were identified for the first time, all from Guangxi. Interestingly, ChPV/TuPV whole-genome sequences from chickens with RSS-like symptoms were more concentrated in Groups C, D and E (Fig. 1d).

**Figure 2:**
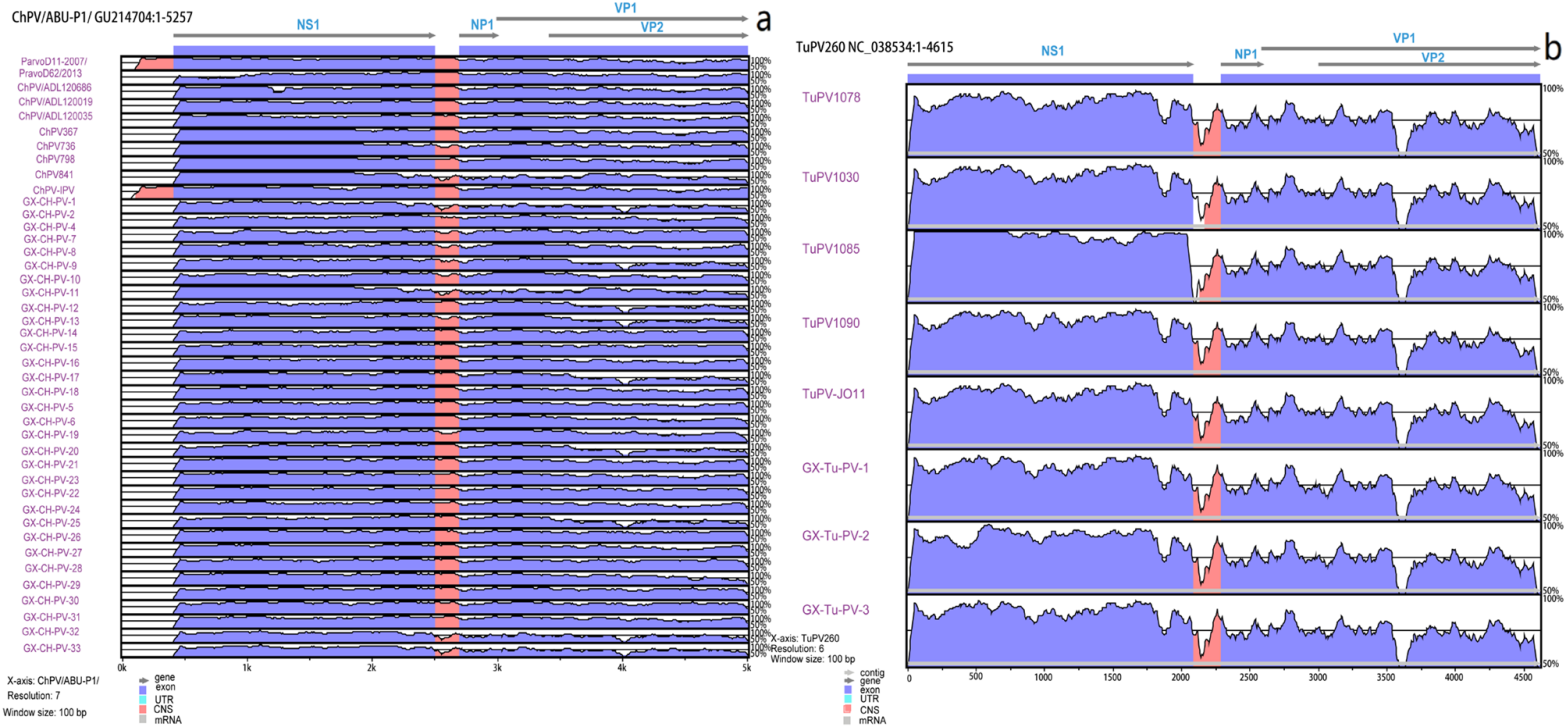
mVISTA whole-genome alignments comparing the nucleotide sequences of the Guangxi ChPV (**a**) and TuPV (**b**) field strains with representative ABU-P1 and 260, 1078, 1030, 1085, 1090 and JO11 TuPV strains. Colour coding: the coloured regions represent a similarity > 90%, and the white regions represent a similarity < 90%. The height of the shaded area at any sampling point is proportional to the genetic relatedness.

The nt alignment of the full genome of the Guangxi ChPV/TuPV strains and 17 reference ChPV/TuPV strains (Fig. 2a,b) revealed conserved and divergent regions between the genomes. Visualizing the genomes in this manner supported the results of the phylogenetic study described above.

### Recombination analysis

Fifteen recombination events were detected in the NS1, VP1 and VP2 genes of 13 Guangxi strains, as shown in Table 2. To further verify the recombination events identified by RDP 5.0, Simplot 3.5.1 software was used to analyse the homology of the recombinant strains. These recombination sequence signals were confirmed by SimPlot analysis (see Supplementary Figs. S6, S7).

**Table 2:** Information on recombination events detected in the genomes of ChPVs/TuPVs.

Serial no.	Strain	Breakpoints		Major parent	Minor parent	Recombinant gene
		Begin	End			
1	GX-CH-PV-16	3192	4610	GX-CH-PV-25	GX-Tu-PV-3	VP2
2	GX-Tu-PV-2	4632	1530	GX-Tu-PV-3	GX-CH-PV-9	NS1
3	GX-CH-PV-24	1703	3046	GX-CH-PV-32	GX-CH-PV-13	NS1, VP1
4	GX-CH-PV-8	3149	4604	GX-CH-PV-14	GX-Tu-PV-3	VP2
5		90	1708	GX-CH-PV-28	GX-CH-PV-18	NS1
6	GX-CH-PV-11	4610	1510	GX-CH-PV-14	GX-Tu-PV-1	NS1
7	GX-CH-PV-4	3174	4580	GX-CH-PV-18	GX-CH-PV-14	VP2
8		792	1306	GX-Tu-PV-2	GX-CH-PV-2	NS1
9	GX-CH-PV-18	4587	3115	GX-CH-PV-21	GX-CH-PV-14	NS1, VP1
10	GX-CH-PV-28	4081	4599	GX-CH-PV-7	GX-CH-PV-24	VP2
11	GX-CH-PV-7	1552	3202	GX-CH-PV-23	GX-CH-PV-9	NS1, VP2
12	GX-CH-PV-6	3235	4554	ChPV-AUB-P1	GX-CH-PV-2	VP2
13	GX-CH-PV-25	1772	4563	GX-CH-PV-20	GX-CH-PV-27	NS1, VP1
14	GX-CH-PV-30	1503	4507	GX-CH-PV-7	GX-CH-PV-13	NS1, VP1
15	GX-CH-PV-22	4591	2548	GX-CH-PV-27	GX-CH-PV-26	NS1

The “Major parent” is the sequence closely related to that from which the greater part of the recombinant’s sequence may have been derived; the “Minor parent” is the sequence closely related to that from which sequences in the proposed recombinant region may have been derived. The actual breakpoint position is undetermined. Most likely, it was overprinted by RDP 5.0.

## Discussion

In the mid-1980s, ChPVs and TuPVs were identified as the causative agents of a pathogenic poultry disease^4,6,10^. Recent genomic characterization studies of the ChPV reference strains ABU-P1, ADL120686, ADL120019, ADL120035, 367, 736, 798, 841, ParvoD62/2013, ParvoD11/2007 and IPV together with the TuPV reference strains 260, 1078, 1030, 1085, 1090 and JO11 have led to an accumulation of genomic sequencing data, providing deeper insights into their molecular features. However, most of the sequence analyses published in the past decade were based on the NS1 and VP genes of ChPVs/TuPVs; few comparative analyses have been based on whole-genome sequences^3,24,35^. These reports have facilitated not only the analysis of the overall genetic architecture of ChPVs/TuPVs but also the development of molecular characterization and diagnostic assays.

No full sequence reports of ChPVs/TuPVs in other Chinese provinces have been published, and in this study, the complete nt sequences of Guangxi ChPV and TuPV strains with or without associations with RSS and PEMS were determined and compared with those of other reference ChPV and TuPV strains at the nt and aa levels. We compared the genomes of 32 ChPV strains and 3 TuPV strains isolated in Guangxi, China, with those of reference ChPV and TuPV strains isolated from the USA, Brazil, Hungary, and South Korea. The nt and aa sequences of the Guangxi ChPV and TuPV strains showed moderate to low similarity to those of the reference ChPV and TuPV strains, with the C-terminal half of the VP2 protein showing the lowest sequence identity. Sequences of ChPV/TuPV strains were compared with those of classical DPV and GPV isolates and showed rather low identity values. Overall, however, these sequencing data suggest that the Guangxi ChPV and TuPV strains, similar to other ChPV and TuPV strains, belong to the genus *Aveparvovirus*. Comparison of ChPV/TuPV isolates from Guangxi and ChPV ABU-P1 strains revealed evidence of selection for the purification of NS1 and VP genes, suggesting that the Chinese ChPV strains evolved independently from the ABU-P1 strain (Hungary).

In the comparison with other parvoviruses, it was found that all VP protein structures were similar. Glycine enrichment may have implications for antigenicity^36^. A study also showed that the leucine residue in VP1(aa 293)/VP2(aa 152) can compress the pores formed by the fivefold cylinder and play an important role in DNA packaging and viral infection^37^. Among the 35 Guangxi ChPVs/TuPVs sequenced in our research, the VP3 start codon was found in 16 strains with reference to the ABU-P1 strain at position 3919–3921 bp, while the remaining 19 strains (including 3 TuPVs) had no VP3 start codon. Thus, the VP3 protein in ChPVs is not generated from ORF2 by alternative splicing. This conclusion is consistent with that of Koo^35^.

Using traditional sequencing methods, we analysed the whole-genome characteristics of 35 ChPV/TuPV strains obtained from Guangxi. Overall, comparing the NS1-, NP1-, and VP-encoding genome segments among the different ChPV and TuPV strains indicated that the regions encoding the outer capsid proteins VP1 (minor capsid protein) and VP2 (major capsid protein) exhibited more variation than the other genes. Specifically, the gene encoding VP2 displayed the greatest sequence divergence, which is reasonable considering that the VP1 and VP2 proteins are components of the outer capsids of ChPVs and TuPVs and may therefore possess several epitopes governing pathogenicity, tissue tropism, and antigenicity^38–41^. Compared with other ChPV/TuPV strains, the Guangxi ChPV and TuPV strains identified in this report may have these properties, and understanding the impact of the nt deletions listed above will require further clarification at the molecular level.

Within genotyping Groups A and B, Guangxi ChPV field strains formed ChPV/TuPV subgroups with the reference ChPV strains, showing high nt similarity to the reference strains. However, the low aa identities (70.6–88.8%) between the subclusters indicate that the Guangxi ChPV field strains in Groups C, D and E are not identical to the reference ChPV strains in ChPV/TuPV Group A, B and F. Similarly, variations in aa identity were also observed between genotyping ChPV/TuPV Groups C, D and E, in which most of the ChPV and TuPV Guangxi field strains formed their own subgroups, distinguishing them from ChPV/TuPV reference strains detected in other countries (e.g., the USA, Brazil, Hungary and South Korea) (Fig. 2). Nonetheless, the novel ChPV/TuPV genotyping Groups C, D and E and emerging variants from the other Guangxi ChPV/TuPV clusters show that ChPV/TuPV has occurred or is continuously undergoing evolutionary mutation or recombination, which should be considered.

Genetic evolution analysis of individual genes and whole genomes based on nucleotide sequences revealed various clustering patterns with reference strains. The topological heterogeneity observed among the phylogenetic trees and sequence alignment indicated that genetic recombination of the VP segment may have occurred between the Guangxi ChPV/TuPV strains and the reference ChPV/TuPV strains. RDP 5.0 and Simplot recombination analysis detected recombination events in 13 strains (13/35, 37.14%) of ChPVs/TuPVs, occurring in the NS1, VP1 and VP2 genes. As shown in the phylogenetic tree of Fig. 1d and Table 2, six of the 13 strains were derived from the novel genotyping groups, indicating that recombination between multiple genotypes of ChPVs/TuPVs may accelerate the emergence of new mutants. These findings suggest that genetic recombination between the Guangxi ChPV and TuPV strains and the reference ChPV/TuPV strains may have played a role in the origination of the Guangxi ChPV and TuPV strains, which is consistent with several reports^7,22^ on ChPV/TuPV genes and with the evolutionary strategies observed in most other species within the Parvoviridae family. This observation suggests that ChPVs and TuPVs might have evolved uniquely in Guangxi over time. However, further studies are needed to corroborate this hypothesis. Collectively, the phylogenetic analysis results provided insights into the origins of the unique genetic configurations observed in the novel Guangxi ChPV/TuPV strains detected in China since 2014.

Based on phylogenetic analysis of the NS1 gene, we hypothesized that the parvoviruses detected in turkey flocks were ChPVs adapted to turkey hosts (TuPV 1085, TuPV 260 and GX-Tu-PV-2). For the VP2 gene, the parvoviruses detected in the turkey flock were ChPVs adapted to a turkey host (TuPV 260); for the VP1 gene and the full genome, the parvoviruses detected in the turkey flock were ChPVs adapted to a turkey host (TuPV 260), while those detected in the chicken flock were TuPVs adapted to a chicken host (GX-Tu-PV-1). Genetic evolution analysis showed that the NS gene was more conserved than the VP1 gene and VP2 gene, and the VP1 gene sequence had the highest degree of differentiation and the largest degree of variation. Therefore, it was speculated that the VP1 gene could replace the whole gene as a genetic marker for the rapid differentiation and classification of ChPVs and TuPVs. Shackelton et al.^27^ also reported that parvovirus has a high atypical mutation rate among DNA viruses, prompting its rapid evolution and host adaptation. Given the epidemiological studies of ChPVs/TuPVs in our laboratory, we suspect that ChPV adaptation to turkeys and TuPV adaptation to chickens are both caused by insufficient disinfection and poor biosafety. In our study, we found that the nt sequences of Guangxi ChPV/TuPV strains showed strong similarity and phylogenetic relationships with the nt sequences of other parvovirus strains isolated from RSS/PEMS cases, which was similar to finding of Zsak et al.^20^, who described the similarity between a TuPV isolate and ChPVs. Therefore, it is possible that some regions of the genome were involved in pathogenicity. Additionally, the detection rate of ChPVs/TuPVs in birds with RSS-like symptoms (91.86%) was higher than that in healthy birds (66.91%), and complicating factors such as mixed or secondary infection with other pathogens may exacerbate the process of parvovirus infection. However, the correlation between sequences and RSS-like symptoms remains to be further studied. Our reports have indicated the presence of variations among Guangxi ChPV and TuPV isolates and incidences of emergence of new isolates worldwide. Whole-genome characterizations of newly emerging Guangxi ChPV and TuPV field strains will provide more detailed insights into ChPV and TuPV mutations and recombination and their relationships with molecular epidemiological features. Therefore, the study of ChPVs/TuPVs in Guangxi will be helpful in tracing the source of the viruses causing epidemics at the molecular level and elucidating the potential transmission route and mode, which is of great significance for epidemiological analysis of disease.

## Materials and methods

### Ethics statement

The present study was approved by the Animal Ethics Committee of the Guangxi Veterinary Research Institute. Sample collections were conducted based on protocol #2019C0406 issued by the Animal Ethics Committee of Guangxi Veterinary Research Institution. All samples were collected from live chickens on approved farms by well-trained veterinarians. All methods were performed in accordance with the relevant guidelines and regulations. In brief, informed consent was obtained from the bird owners, and biological samples were gently collected from the chickens and turkeys using sterilized cotton swabs. The birds were not anaesthetized before sampling, and the sampled birds were observed for 30 min after sampling before they were returned to their cages. All sections of this study adhere to ARRIVE guidelines for reporting animal research.

### Sample collection

The ChPV and TuPV field strains used in this study were obtained from commercial chicken and turkey flocks, including both clinically healthy and suspected RSS/PEMS-affected birds. A total of 1526 throat and cloacal swab samples were collected from chickens and turkeys from Liuzhou, Guilin, Fangchenggang, Hechi, Chongzuo, Qinzhou, Yulin, Beihai, Nanning and Wuzhou cities in Guangxi, southern China, from 2014 to 2022. All samples were processed according to the protocol of the World Organization for Animal Health (OIE). For more details, please refer to https://www.oie.int/fileadmin/Home/eng/Health_standards/tahm/1.01.02_COLLECTION_DIAG_SPECIMENS.pdf.

### DNA extraction, genome-segment amplification and nucleotide sequencing

The presence of ChPV/TuPV in the throat and cloacal swab samples was detected by PCR^17,20^. Information on the detection primers 561-bp NS1 and 249-bp VP1/VP2 is listed in Supplementary Table S3. By referring to the complete sequences of 3 prototype ChPV and TuPV strains from GenBank, three specific primer pairs (see Supplementary Table S4) were designed to amplify the complete ChPV and TuPV genomes of 32 positive samples and 3 positive samples, respectively.

### Sequence analysis

Sanger sequence assembly and nt sequence translation were performed using DNASTAR Lasergene 7.1. The ORF was predicted on the NCBI website (http://www.ncbi.nlm.nih.gov/gorf/gorf.html). Sequence similarity was assessed by NCBI BLAST search and using DNAMAN version 10 software (Lynnon Biosoft). Sequence alignment was performed using the ClustalW 2.1 program (http://www.clustal.org/clustal2/#Download). Neighbour-joining trees were generated using the MEGA (version 11) program (https://www.megasoftware.net/), and bootstrap analysis was performed to verify the tree topology using absolute distances following 1000 bootstrap replicates^42^. The mVISTA online platform was used for ChPV/TuPV genome-wide comparative analysis (http://genome.lbl.gov/vista/mvista/submit.shtml). Sequence recombination analysis of the NS1, VP1 and VP2 genes of 35 Guangxi ChPV/TuPV strains and 17 reference ChPV/TuPV strains was performed using RDP 5.0 and Simplot 3.5.1. To ensure the consistency and accuracy of the results, 7 different recombination analysis methods were used for analysis. For example, more than 4 analysis methods showed the presence of recombination events, and at a P value < 10^–6^, the recombination event was judged to be credible^43^.

### Supplementary Information

Supplementary Information.

## Data Availability

The datasets generated and analysed during the current study are available in the NCBI genome repository or from the corresponding author upon reasonable request. The accession numbers are as follows: GX-CH-PV-1 (KX084399), GX-CH-PV-2 (KX084400), GX-CH-PV-4 (KX084401), GX-CH-PV-5 (KX133426), GX-CH-PV-6 (KX133427), GX-CH-PV-7 (KU523900), GX-CH-PV-8 (KX133415), GX-CH-PV-9 (KX133416), GX-CH-PV-10 (KX133417), GX-CH-PV-11 (KX133418), GX-CH-PV-12 (KX133419), GX-CH-PV-13 (KX133420), GX-CH-PV-14 (KX133421), GX-CH-PV-15 (KX133422), GX-CH-PV-16 (KX133423), GX-CH-PV-17 (KX133424), GX-CH-PV-18 (KX133425), GX-CH-PV-19 (MG602509), GX-CH-PV-20 (MG602510), GX-CH-PV-21 (MG602511), GX-CH-PV-22 (MG602512), GX-CH-PV-23 (MG602513), GX-CH-PV-24 (MG602514), GX-CH-PV-25 (MG602515), GX-CH-PV-26 (MG602516), GX-CH-PV-27 (MG602517), GX-CH-PV-28 (MG602518), GX-CH-PV-29 (MG602519), GX-CH-PV-30 (MG602520), GX-CH-PV-31 (OQ437199), GX-CH-PV-32 (OQ437200), GX-CH-PV-33 (OQ437201), GX-Tu-PV-1 (KX084396), GX-Tu-PV-2 (KX084397), GX-Tu-PV-3 (KX084398) and GX-Tu-PV-3 (KX084398).
