# Supplementary material for: Molecular characterization of emerging chicken and turkey parvovirus variants and novel strains in Guangxi, China
Source: Sci Rep. 2023 Aug 11;13:13083. doi: 10.1038/s41598-023-40349-5 (PMC10421884; doi:10.1038/s41598-023-40349-5)
Supplement: Supplementary file 1 — Supplementary Information. [file 41598_2023_40349_MOESM1_ESM.docx]

s

sss

**Supporting Information**

**Molecular characterization of emerging chicken and turkey parvovirus variants and novel strains in Guangxi, China**

**Yanfang Zhang^1, 2,^^[[1]](#footnote-1)^#, Bin Feng^1, 2,^^[[2]](#footnote-2)^#, Zhixun Xie^1, 2,^^[[3]](#footnote-3)^*^,^ ✉, Minxiu Zhang^1, 2^, Qing Fan^1, 2^, Xianwen Deng^1, 2^, Zhiqin Xie^1, 2^, Meng Li^1, 2^, Tingting Zeng^1, 2^, Liji Xie^1, 2^, Sisi Luo^1, 2^, Jiaoling Huang^1, 2^ & Sheng Wang^1, 2^**

^1^Guangxi Key Laboratory of Veterinary Biotechnology, Guangxi Veterinary Research Institute, Nanning 530000, Guangxi, China

^2^Key Laboratory of China (Guangxi)-ASEAN Cross-border Animal Disease Prevention and Control, Ministry of Agriculture and Rural Affairs of China, Nanning 530000, Guangxi, China

^#^ Yanfang Zhang and Bin Feng have contributed equally as the first authors.*Correspondence: Zhixun Xie. ✉E-mail: [xiezhixun@126.com](mailto:xiezhixun@126.com).

Figure

Figure

**
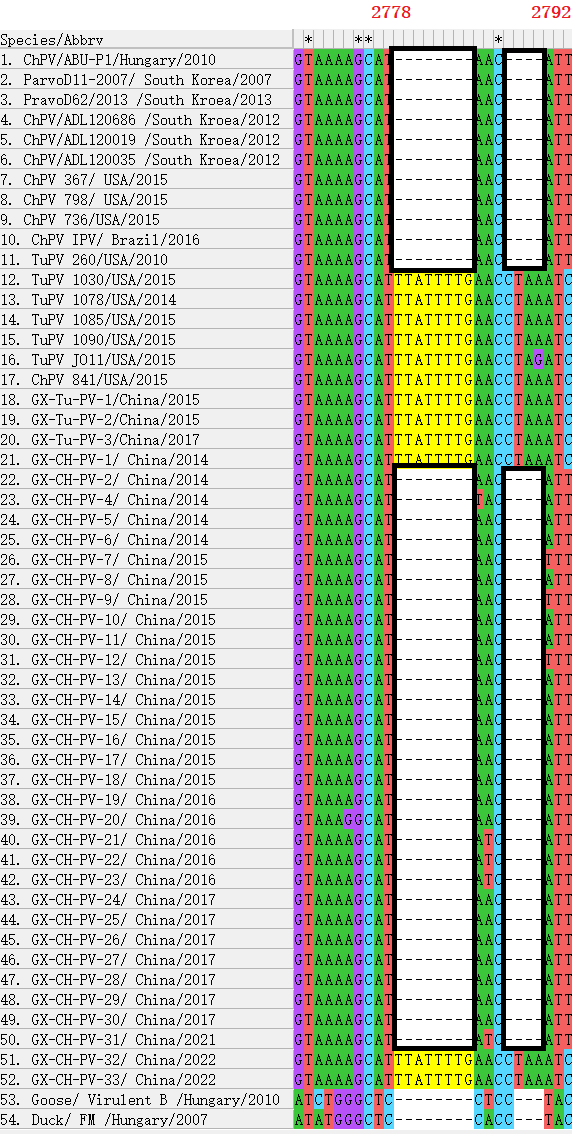
**

**Fig. S1** The nucleotide (nt) deletions in the genomes of the studied ChPV and TuPV strains. An eight-nt (TTATTTTG) deletion and a four-nt (CTAA) deletion (corresponding to nt 2778 to 2785 and nts 2789 to 2792 in the NP1 gene of strain ABU-P1, respectively) were observed in the ChPV and TuPV strains;

**
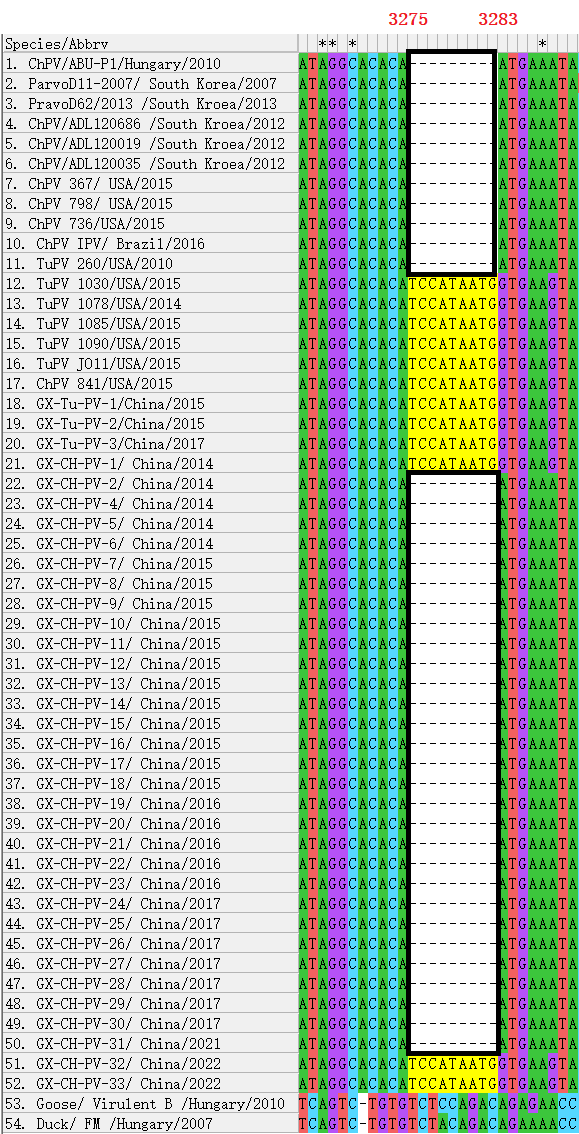
**

**Fig. S2**  The nucleotide (nt) deletions in the genomes of the studied ChPV and TuPV strains. A nine-nt (TCCATAATG) deletion (corresponding to nt 3275 to 3283 in the VP1 gene of strain ABU-P1) was found in the ChPV and TuPV strains;

**
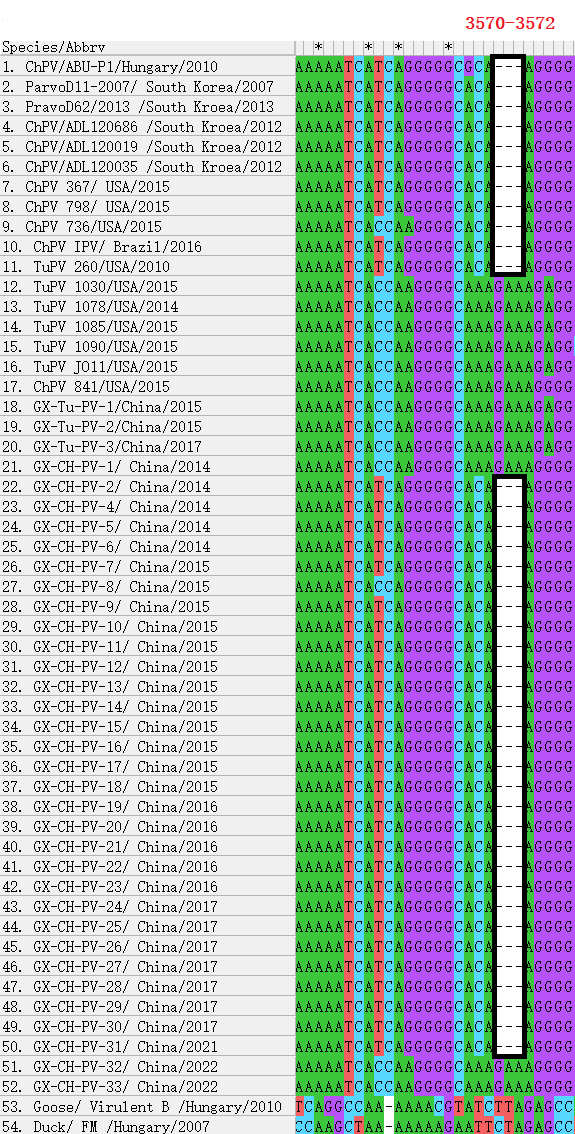
**

**Fig. S3** The nucleotide (nt) deletions in the genomes of the studied ChPV and TuPV strains. A three-nt (GAA) deletion (corresponding to nt 3570 to 3572 in the VP2 gene of strain ABU-P1) was observed in the ChPV and TuPV strains. The nt positions are based on the ABU-P1 strain.

**
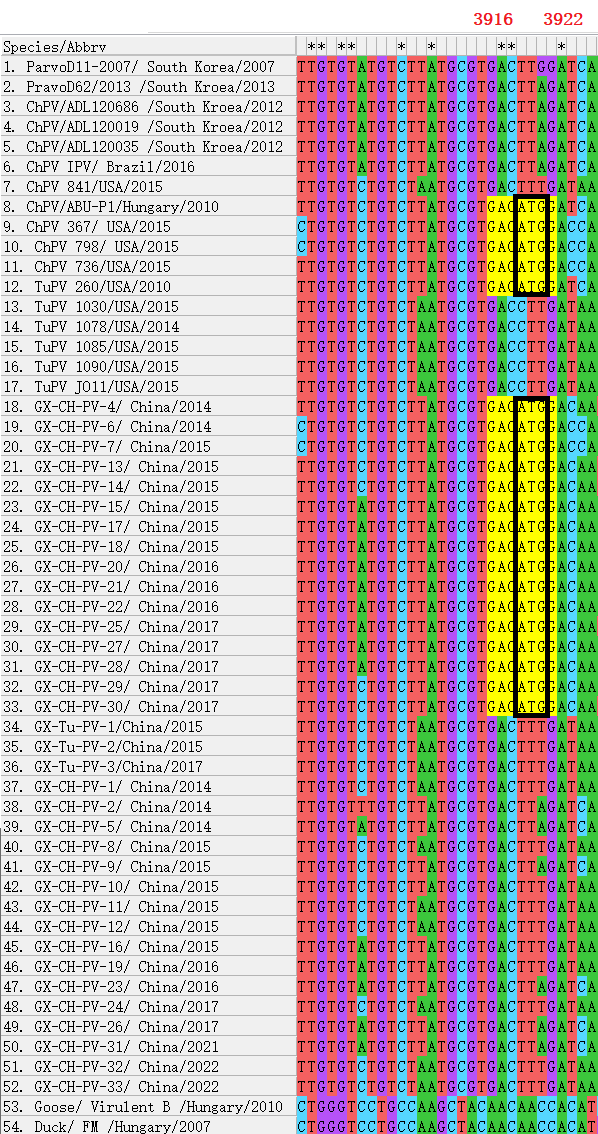
**

**Fig. S4**  Putative VP3 start codons among different ChPV and TuPV strains. The base marked with colour in the black box represents the starting codon of the identified VP3 ORF, which is located at the position of the reference sequence ABU-P1 nucleotide 3919-3921.

**
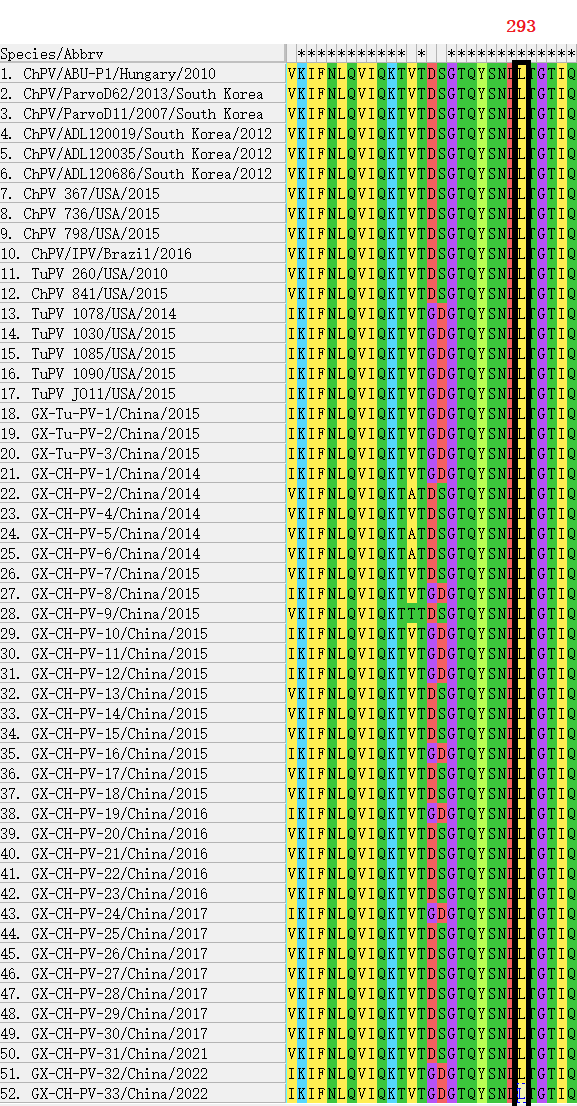
**

**Fig. S5**  Structural motifs in the VP genes of the ChPV and TuPV strains. The aa positions are based on the ABU-P1 strain. The box in black represents the structure motif composed of fivefold cylinder regions. The conserved leucine at position VP1 293 in the red box represents the structural motif of the cylindrically protruded pore contraction at each fivefold axis of symmetry.

**
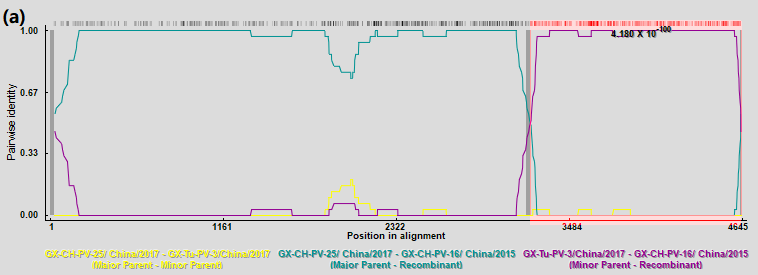

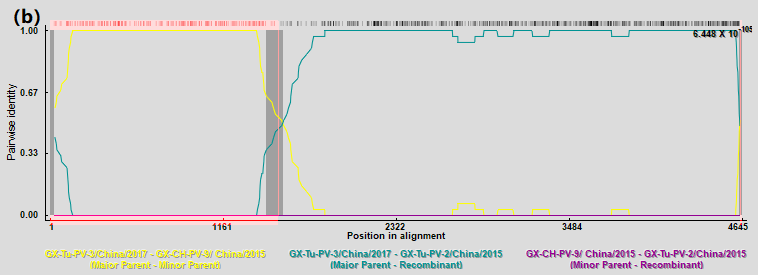

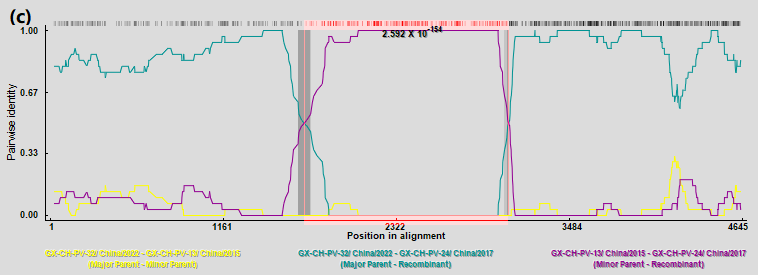

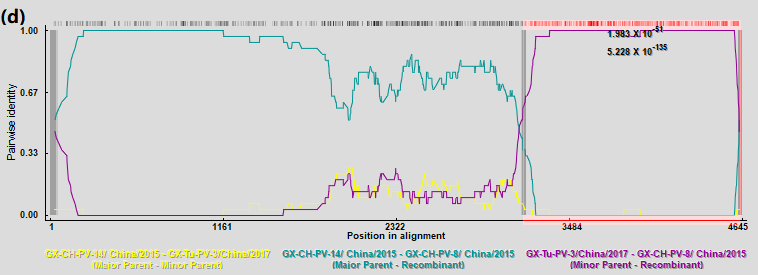

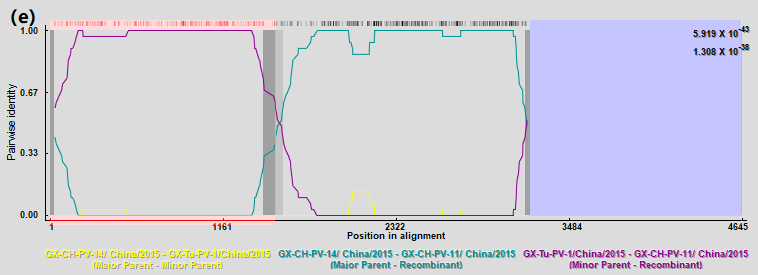

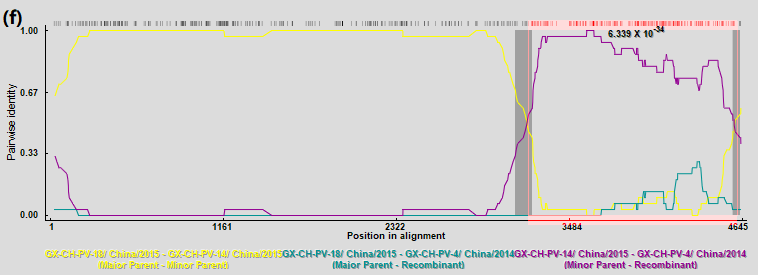

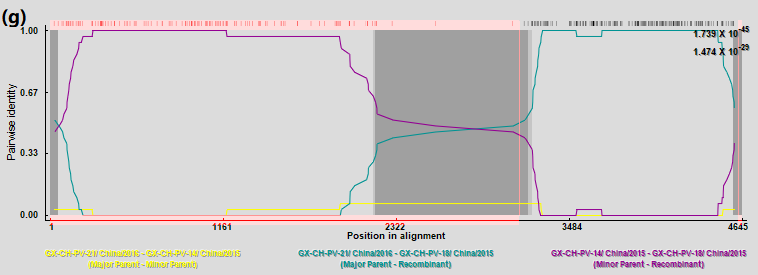

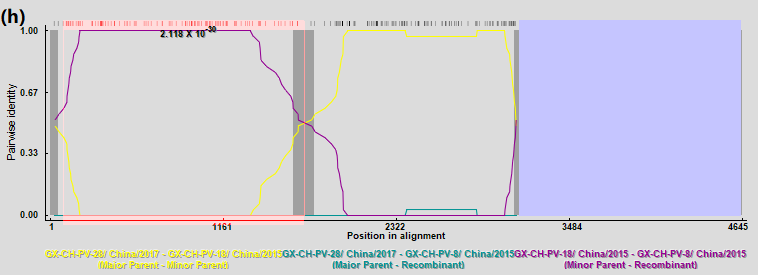

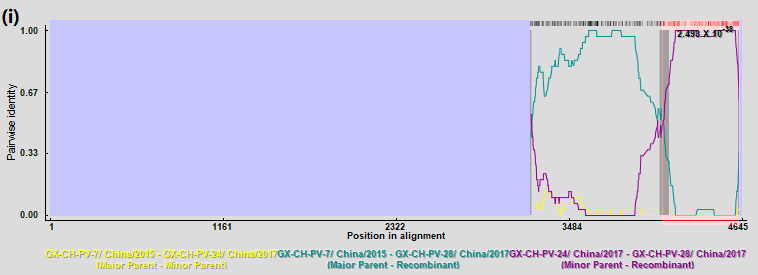

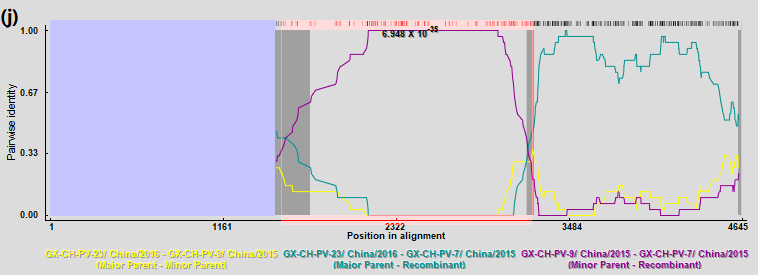

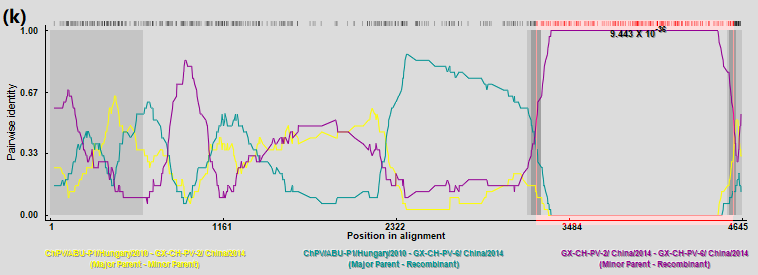

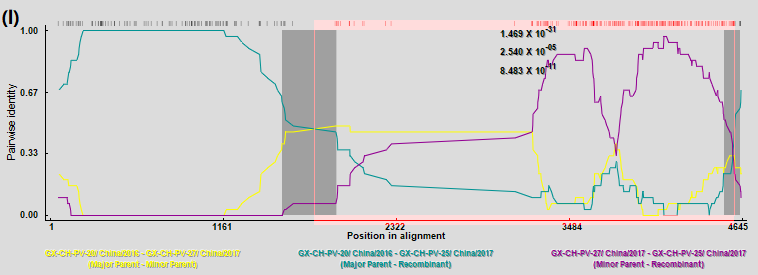

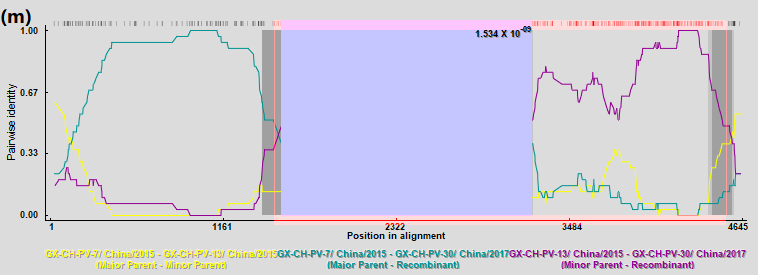

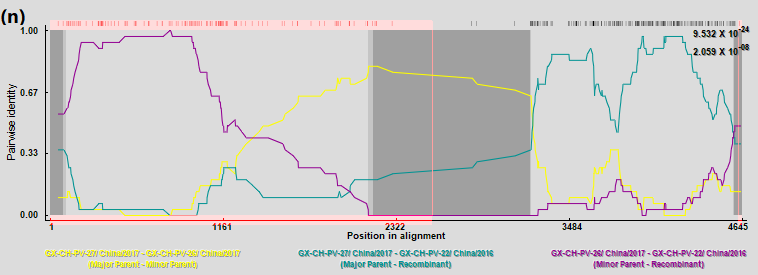

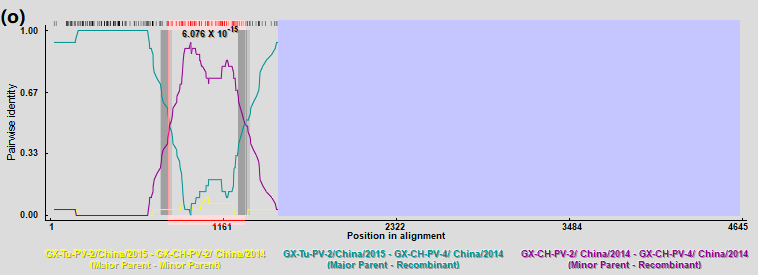
**

**Fig. S6**  Recombination analysis resultsof the ChPV/TuPV isolates using RDP5.0. **(a)~(o)** correspond to the sequence in Table 2.


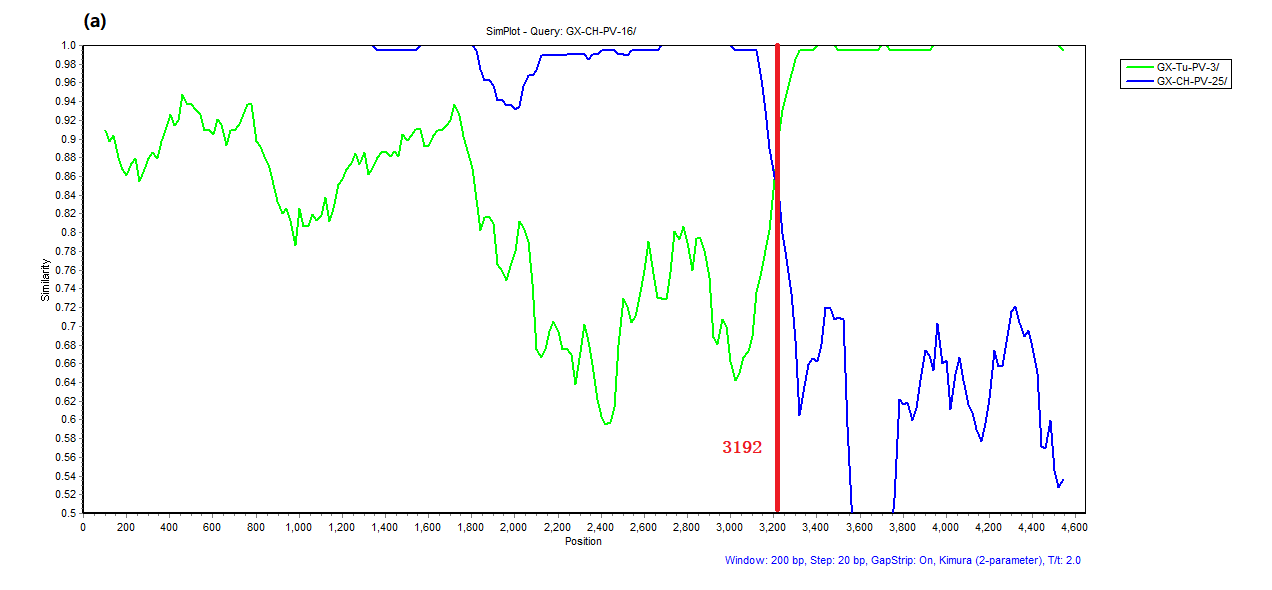

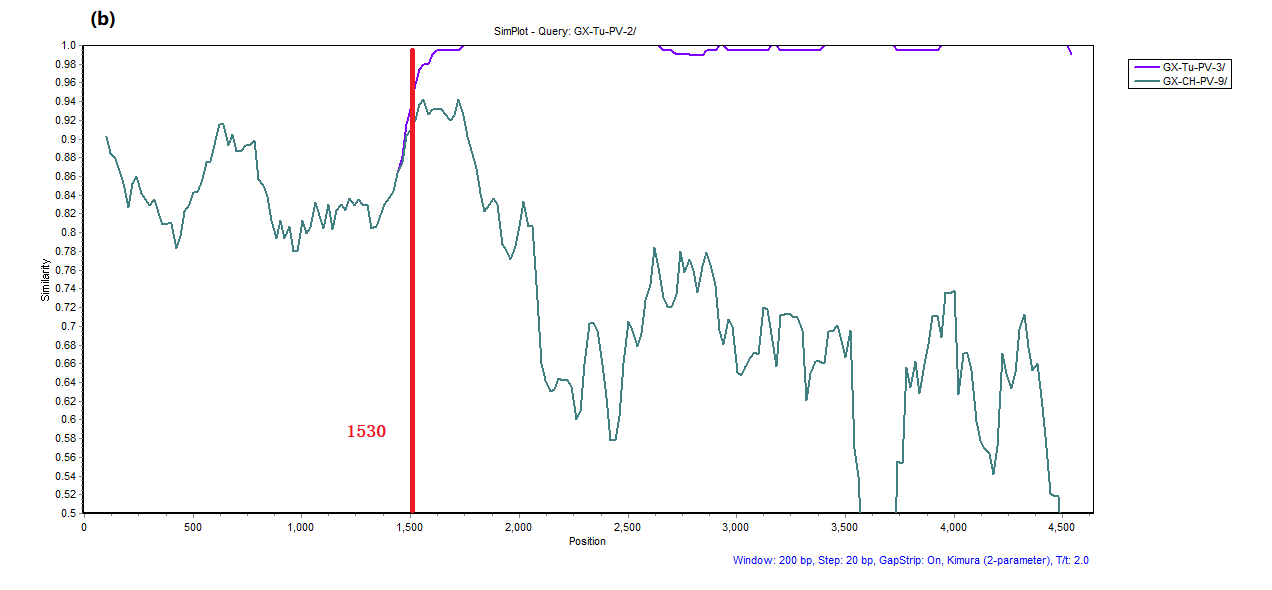

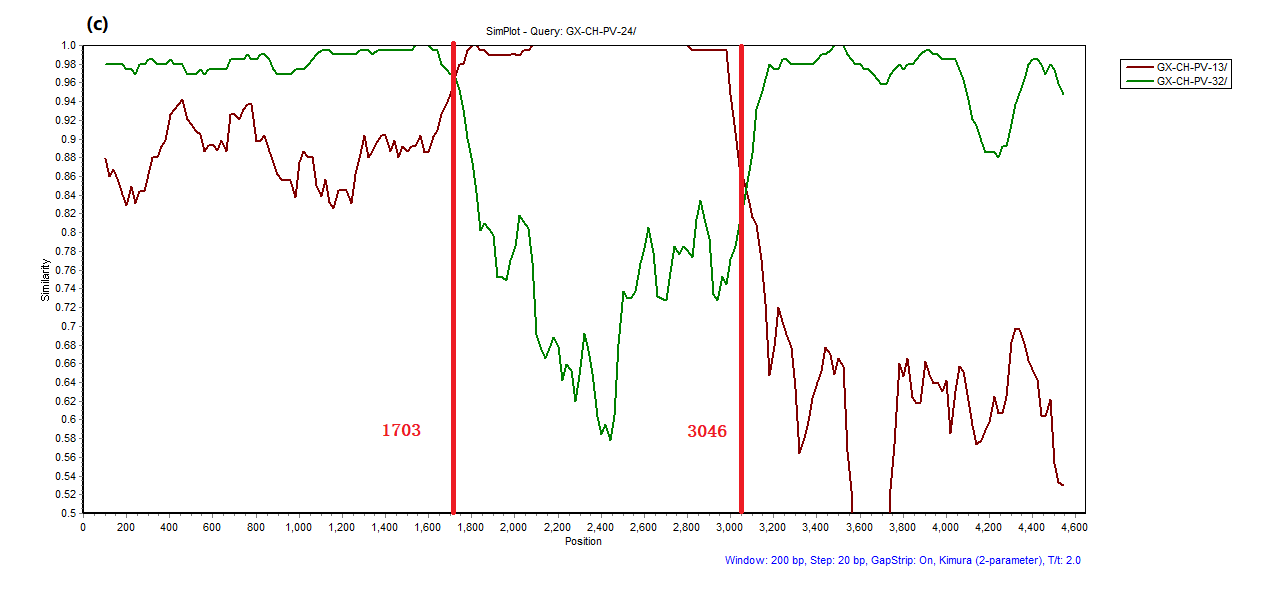

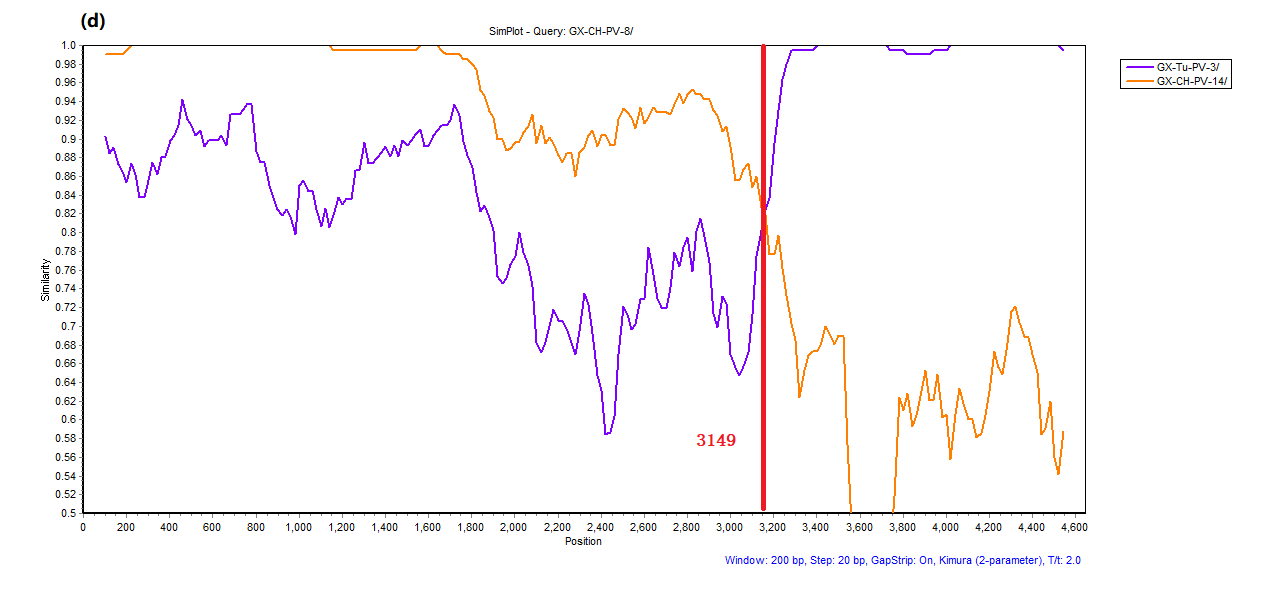

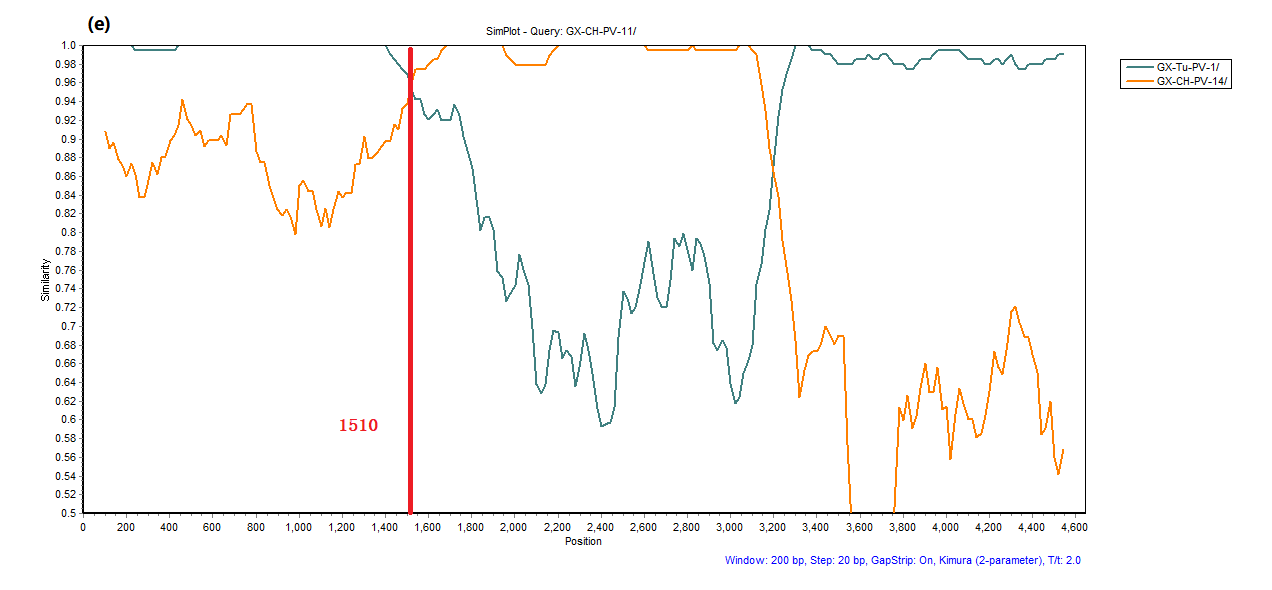

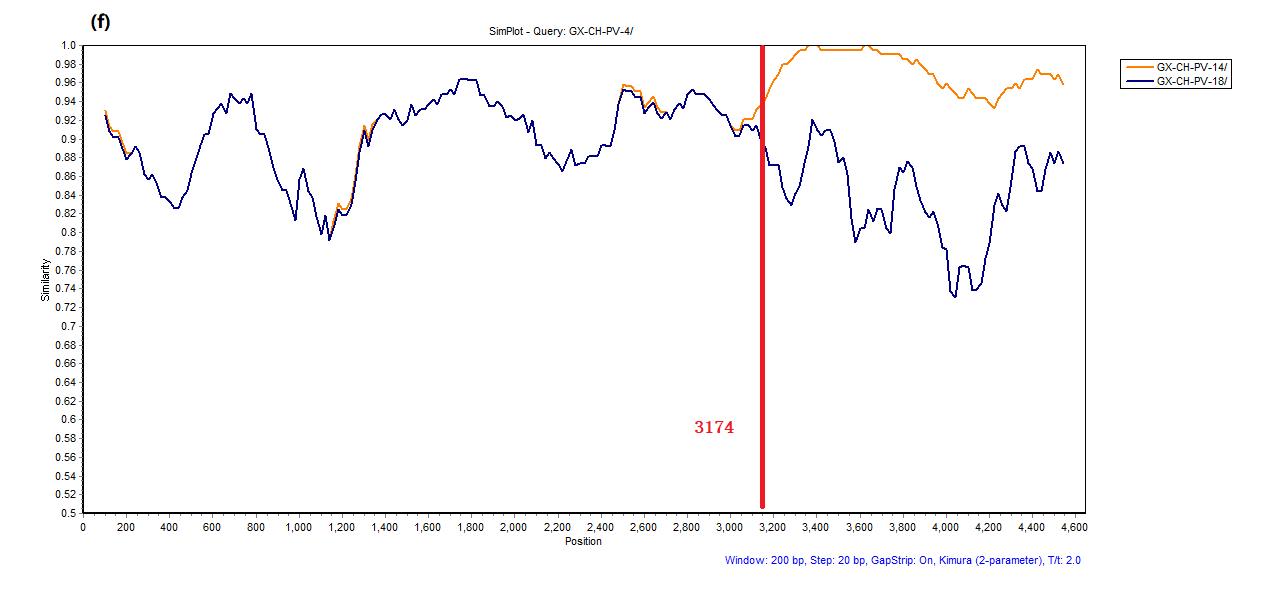

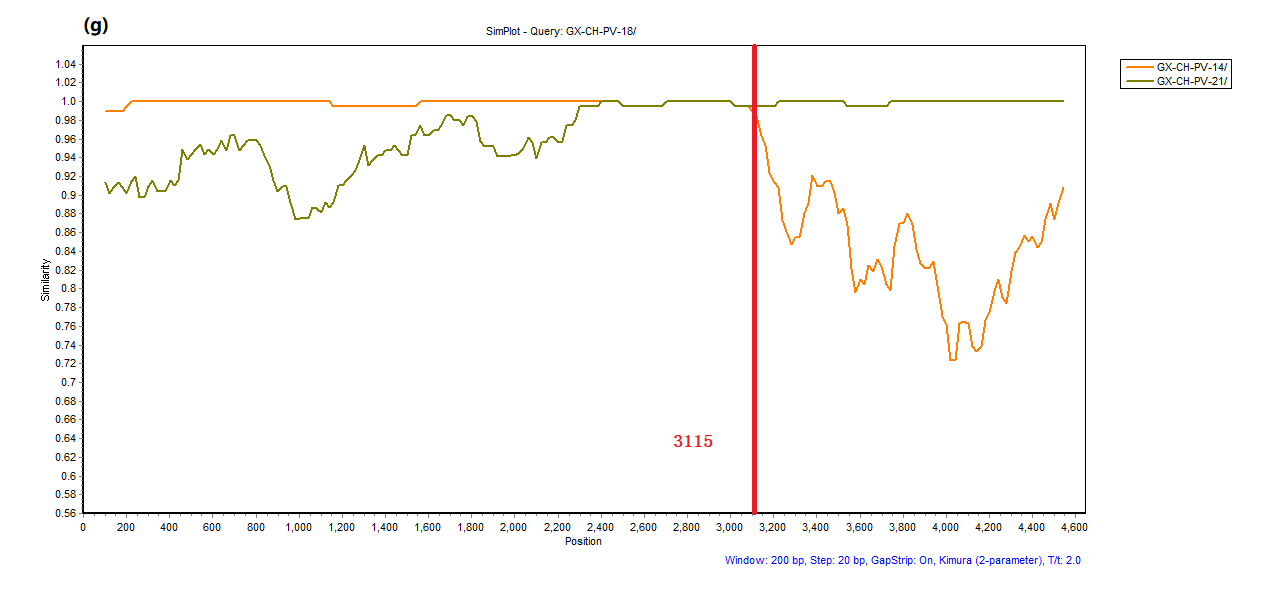

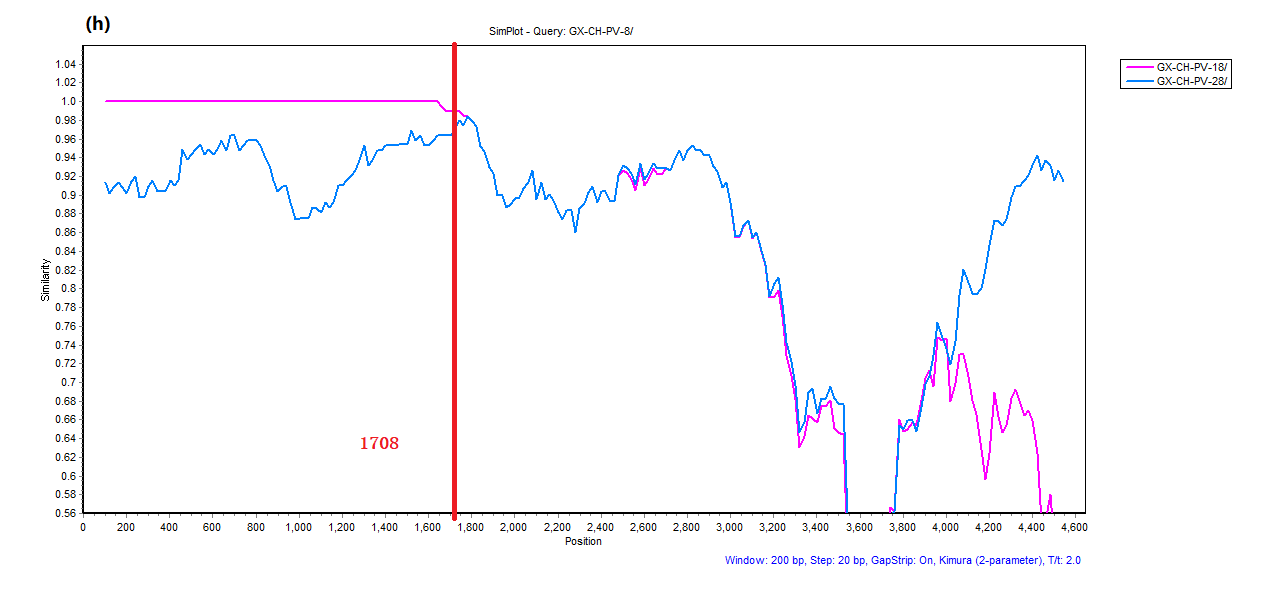

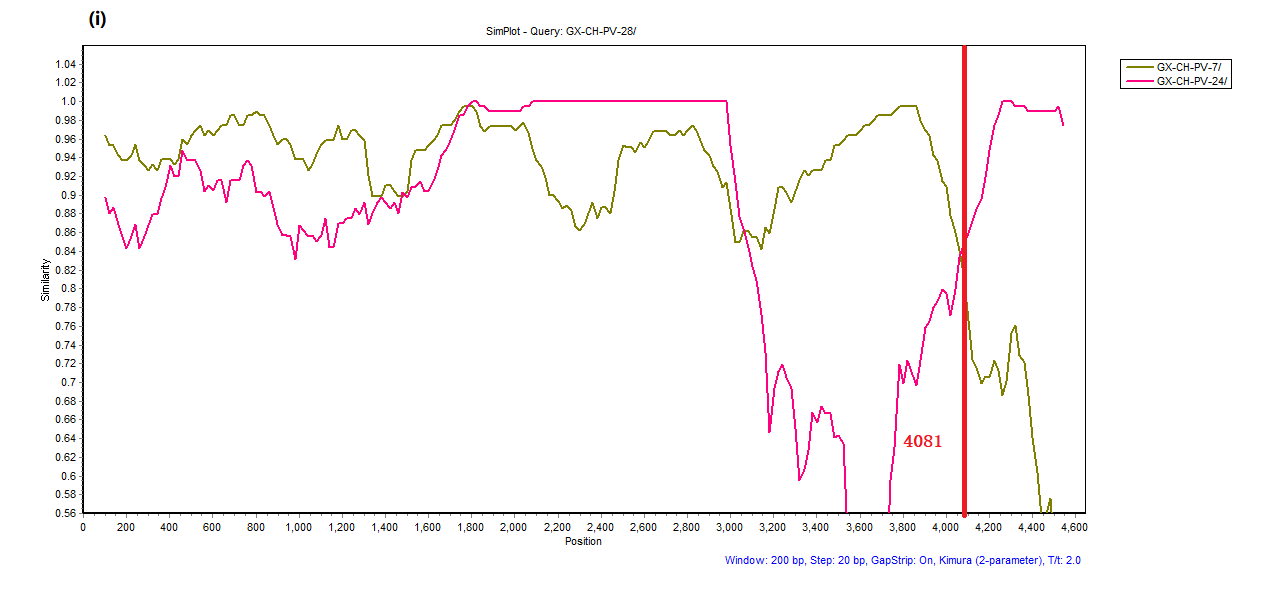

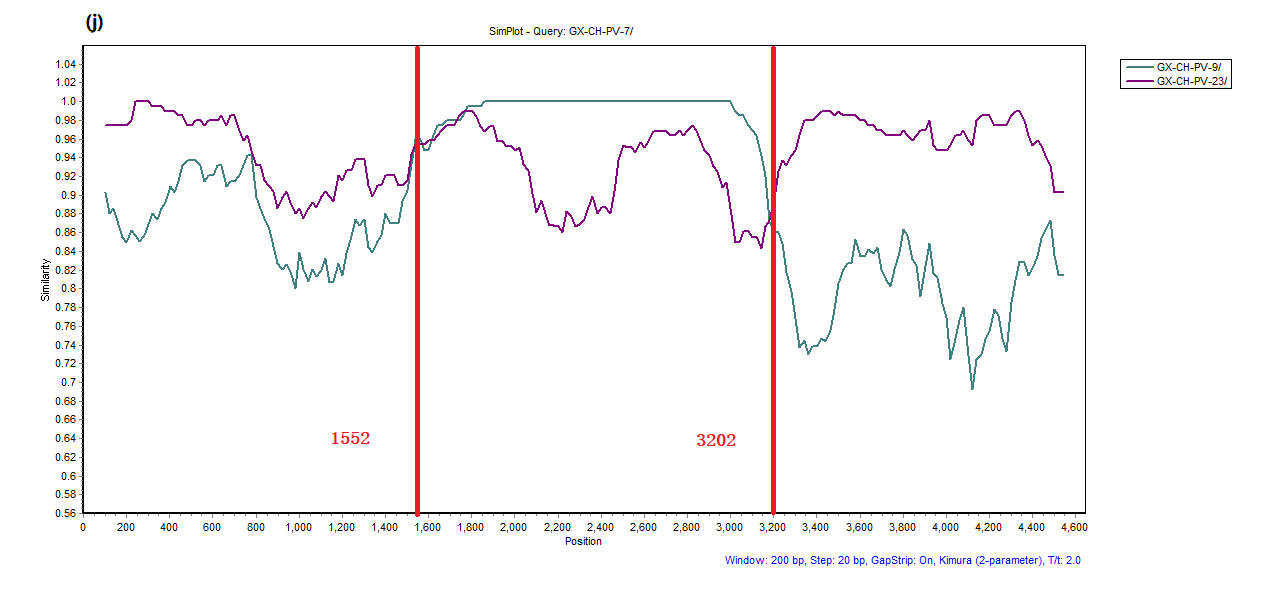

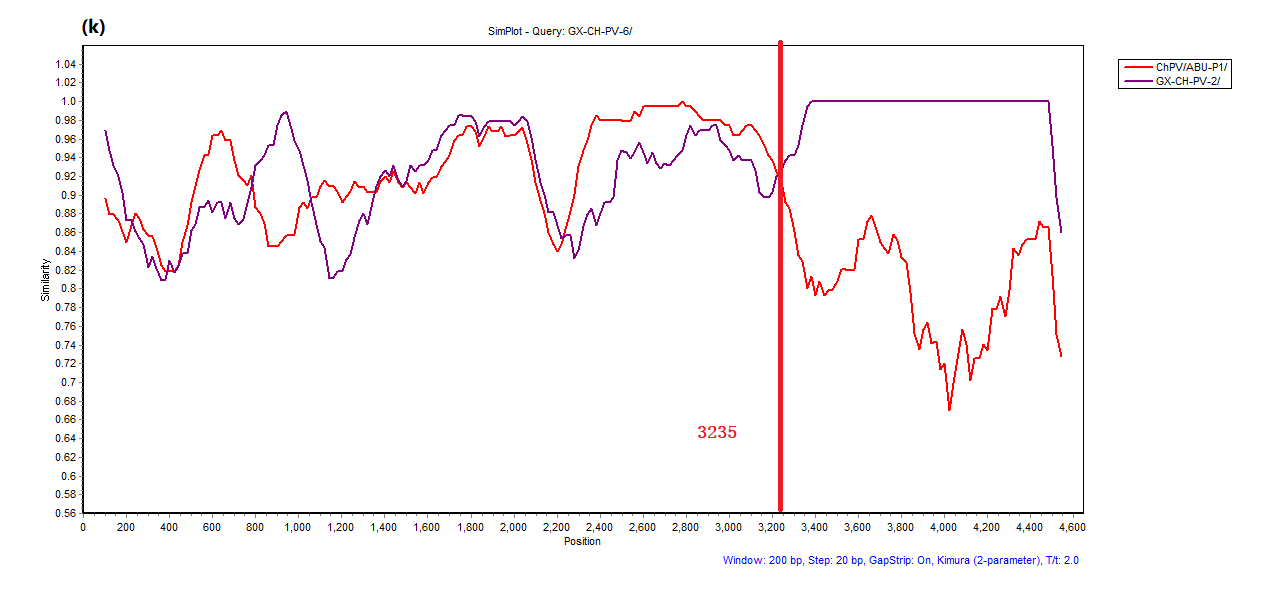

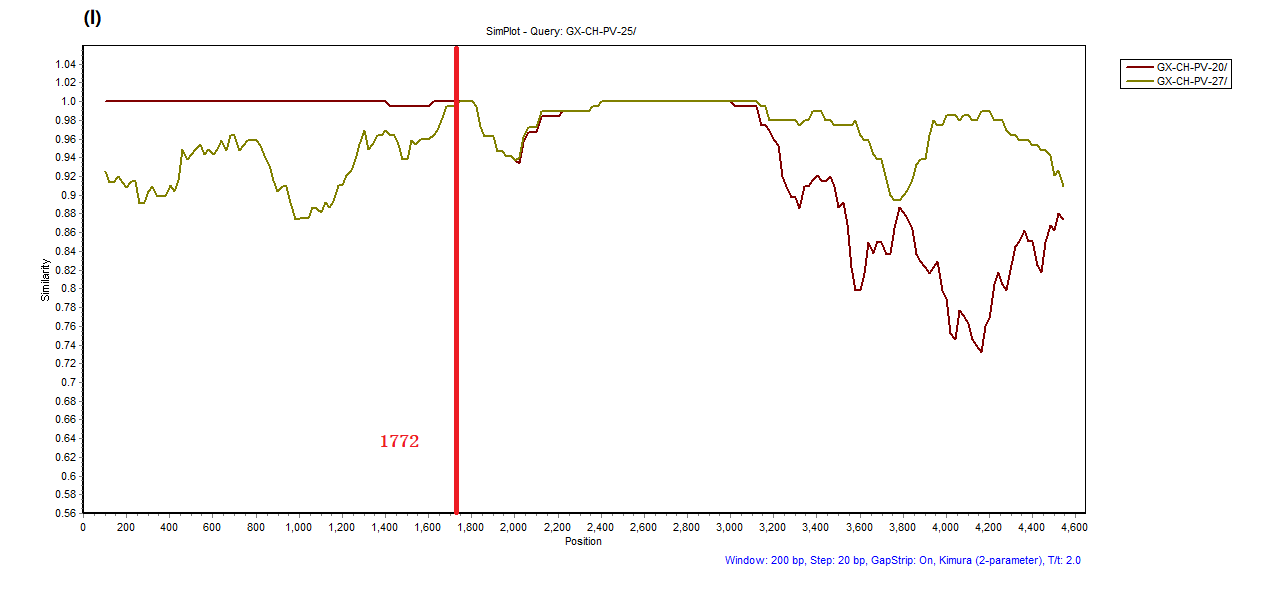

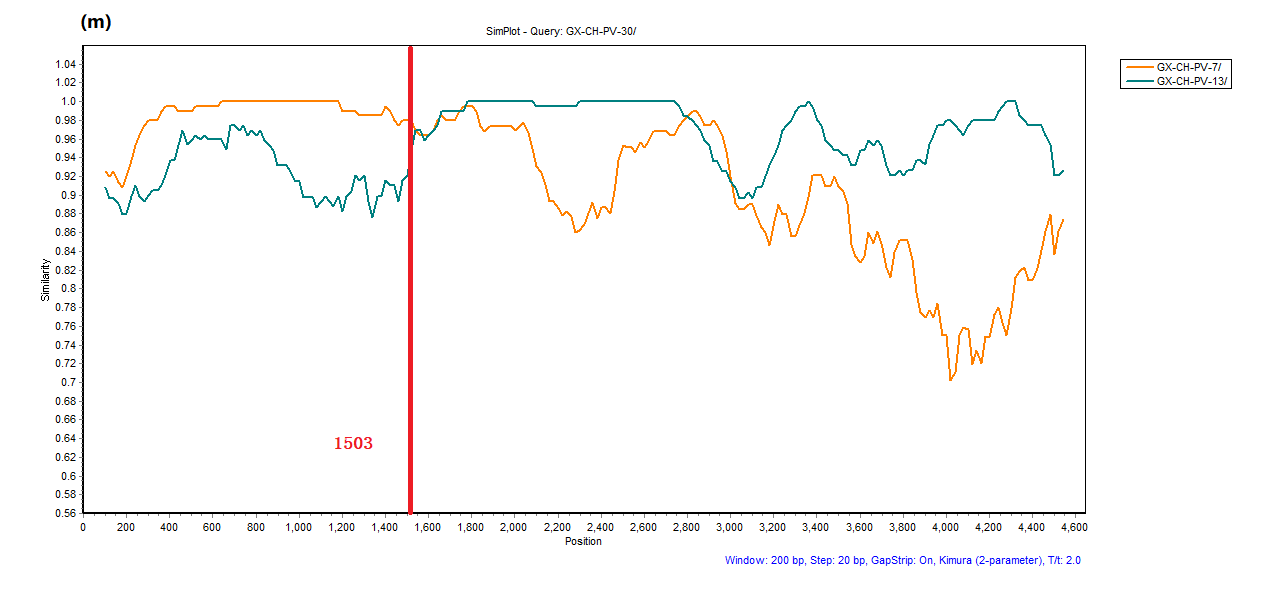

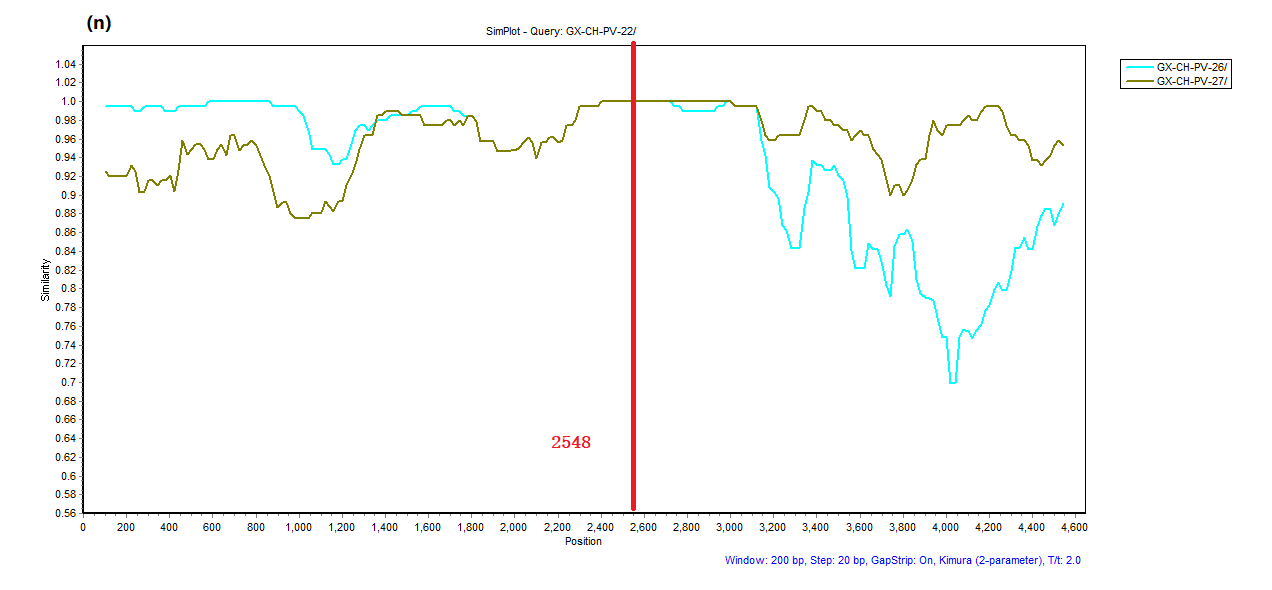

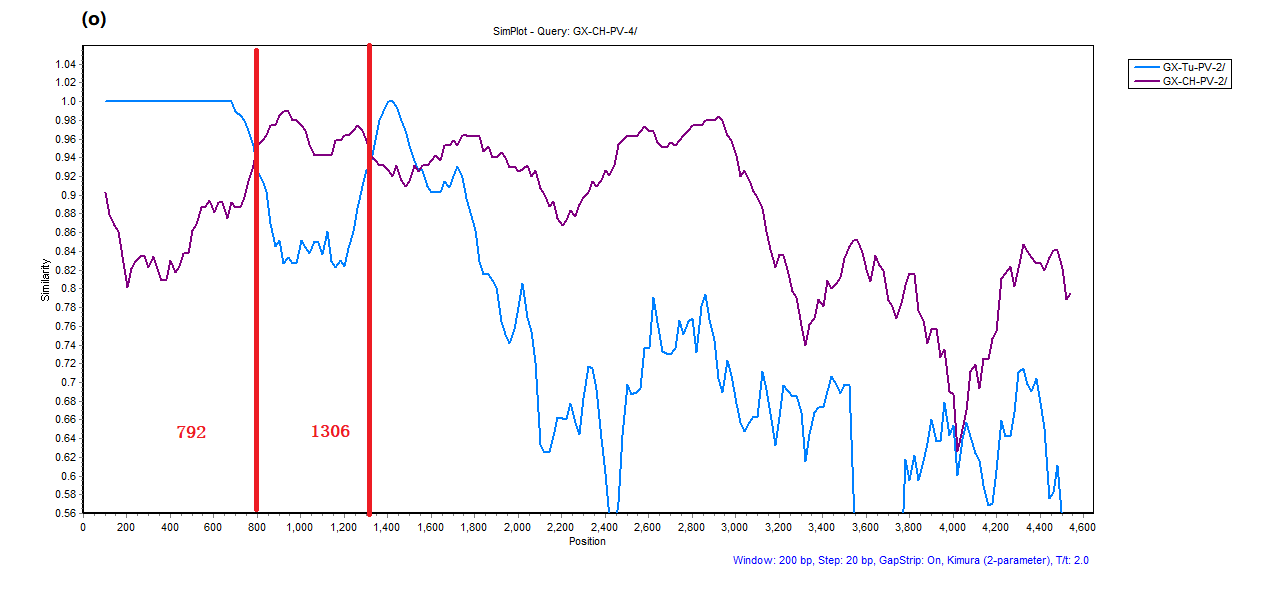


**Fig. S7**  Recombination analysis results **(a)~(o)** of the ChPV/TuPV isolates using Simplot 3.5.1. **(a)~(o)** correspond to the sequence in Table 2.

**Table S1 Similarity of genes among 35 Guangxi ChPV/TuPV and reference strains**

| **Strain** | **Genome segment** | **Size (bp)** | **Protein size (aa)** | **Encoded**  **protein** | **Lowest similarity(%) to strain in GenBank** | **Highest similarity(%) to strain in GenBank** |
| --- | --- | --- | --- | --- | --- | --- |
| GX-CH-PV-1 | Full genome | 4642 | — | — | 83 (TuPV JO11,KM598421) | 90 (ParvoD11/2007, KM254173) |
|  | NS1 | 2088 | 696 | NS1 | 86 (ParvoD11/2007, KM254173) | 94 (ChPV 841, KM598417) |
|  | NP1 | 311 | 103 | NP1 | 75 (ChPV ABU-P1, GU214704) | 99 (ChPV 841, KM598417) |
|  | VP1 | 2034 | 678 | VP1 | 74 (ChPV ABU-P1, GU214704) | 93 (TuPV 1030, KM598418) |
|  | VP2 | 1611 | 537 | VP2 | 73 (ChPV ABU-P1, GU214704) | 92 (TuPV 1030, KM598418) |
| GX-CH-PV-4 | Full genome | 4612 | — | — | 81 (TuPV JO11,KM598421) | 93 (TuPV 260, GU214706) |
|  | NS1 | 2085 | 695 | NS1 | 87 (ParvoD11/2007, KM254173) | 91 (ChPV 367, KM598414) |
|  | NP1 | 305 | 101 | NP1 | 72 (TuPV JO11,KM598421) | 95 (ChPV 736, KM598415) |
|  | VP1 | 2028 | 676 | VP1 | 74 (TuPV 1090, KM598420) | 95 (TuPV 260, GU214706) |
|  | VP2 | 1611 | 537 | VP2 | 73 (TuPV 1090, KM598420) | 95 (TuPV 260, GU214706) |
| GX-CH-PV-5 | Full genome | 4615 | — | — | 81 (TuPV JO11,KM598421) | 91(ChPV ADL120035, KJ486490) |
|  | NS1 | 2086 | 695 | NS1 | 88 (TuPV JO11,KM598421) | 94 (ChPV ADL120035, KJ486490) |
|  | NP1 | 305 | 101 | NP1 | 75 (TuPV JO11,KM598421) | 99 (ChPV IPV, KU569162) |
|  | VP1 | 2029 | 676 | VP1 | 75 (TuPV 1090, KM598420) | 89 (ParvoD11/2007, KM254173) |
|  | VP2 | 1611 | 537 | VP2 | 74 (TuPV 1090, KM598420) | 88 (ParvoD11/2007, KM254173) |
| GX-CH-PV-6 | Full genome | 4615 | — | — | 81 (TuPV JO11, KM598421) | 90 (ChPV ABU-P1, GU214704) |
|  | NS1 | 2086 | 695 | NS1 | 88 (TuPV JO11, KM598421) | 92 (ChPV 367, KM598414) |
|  | NP1 | 305 | 101 | NP1 | 75 (TuPV JO11,KM598421) | 99 (ChPV ABU-P1, GU214704) |
|  | VP1 | 2028 | 676 | VP1 | 74 (TuPV 1090, KM598420) | 89 (ParvoD11/2007, KM254173) |
|  | VP2 | 1611 | 537 | VP2 | 74 (TuPV 1090, KM598420) | 88 (ParvoD11/2007, KM254173) |
| GX-CH-PV-7 | Full genome | 4612 | — | — | 81 (TuPV JO11, KM598421) | 94 (ChPV IPV, KU569162) |
|  | NS1 | 2085 | 695 | NS1 | 88 (TuPV JO11, KM598421) | 97 (ChPV IPV, KU569162) |
|  | NP1 | 305 | 101 | NP1 | 75 (TuPV JO11,KM598421) | 95 (ChPV 736, KM598415) |
|  | VP1 | 2028 | 676 | VP1 | 73 (TuPV 1090, KM598420) | 93 (ChPV 367, KM598414) |
|  | VP2 | 1611 | 537 | VP2 | 72 (TuPV 1090, KM598420) | 92 (ChPV 367, KM598414) |
| GX-CH-PV-8 | Full genome | 4615 | — | — | 85 (ParvoD11/2007, KM254173) | 88 (TuPV 1030, KM598418) |
|  | NS1 | 2085 | 695 | NS1 | 88 (TuPV JO11, KM598421) | 94 (ChPV 367, KM598414) |
|  | NP1 | 305 | 101 | NP1 | 74 (TuPV JO11, KM598421) | 95 (TuPV 260, GU214706) |
|  | VP1 | 2028 | 676 | VP1 | 78 (ChPV 367, KM598414) | 93 (TuPV 1030, KM598418) |
|  | VP2 | 1611 | 537 | VP2 | 74 (ChPV 367, KM598414) | 96 (TuPV JO11, KM598421) |
| GX-CH-PV-9 | Full genome | 4612 | — | — | 83 (ChPV 841, KM598417) | 90 (TuPV 260, GU214706) |
|  | NS1 | 2085 | 695 | NS1 | 88 (ParvoD11/2007, KM254173) | 96 (TuPV 1090, KM598420) |
|  | NP1 | 305 | 101 | NP1 | 75 (TuPV JO11, KM598421) | 95 (ChPV 736, KM598415) |
|  | VP1 | 2028 | 676 | VP1 | 74 (TuPV 1090, KM598420) | 88 (ParvoD11/2007, KM254173) |
|  | VP2 | 1611 | 537 | VP2 | 73 (TuPV 1090, KM598420) | 86 (ParvoD11/2007, KM254173) |
| GX-CH-PV-10 | Full genome | 4616 | — | — | 84 (ChPV 841, KM598417) | 91 (TuPV 1030, KM598418) |
|  | NS1 | 2085 | 695 | NS1 | 88 (ParvoD11/2007, KM254173) | 95 (TuPV 1090, KM598420) |
|  | NP1 | 305 | 101 | NP1 | 74 (TuPV JO11, KM598421) | 99 (ChPV IPV, KU569162) |
|  | VP1 | 2029 | 676 | VP1 | 79 (ChPV ABU-P1, GU214704) | 92 (TuPV JO11, KM598421) |
|  | VP2 | 1612 | 537 | VP2 | 75 (ChPV ABU-P1, GU214704) | 96 (TuPV JO11, KM598421) |
| GX-CH-PV-11 | Full genome | 4613 | — | — | 84 (ChPV 841, KM598417) | 91(TuPV 1030, KM598418) |
|  | NS1 | 2086 | 695 | NS1 | 88 (ParvoD11/2007, KM254173) | 95 (TuPV 1090, KM598420) |
|  | NP1 | 305 | 101 | NP1 | 74 (TuPV JO11, KM598421) | 99 (ChPV IPV, KU569162) |
|  | VP1 | 2027 | 675 | VP1 | 79 (ChPV ABU-P1, GU214704) | 93 (TuPV JO11, KM598421) |
|  | VP2 | 1611 | 537 | VP2 | 74 (ChPV ABU-P1, GU214704) | 96 (TuPV JO11, KM598421) |
| GX-CH-PV-12 | Full genome | 4612 | — | — | 86 (ChPV 841, KM598417) | 89 (TuPV 260, GU214706) |
|  | NS1 | 2085 | 695 | NS1 | 88 (TuPV JO11, KM598421) | 96 (TuPV 260, GU214706) |
|  | NP1 | 305 | 101 | NP1 | 75 (TuPV JO11, KM598421) | 95 (ChPV 736, KM598415) |
|  | VP1 | 2028 | 676 | VP1 | 79 (ChPV 367, KM598414) | 93 (TuPV JO11, KM598421) |
|  | VP2 | 1611 | 537 | VP2 | 74 (ChPV 367, KM598414) | 96 (TuPV JO11, KM598421) |
| GX-CH-PV-13 | Full genome | 4614 | — | — | 81(TuPV JO11, KM598421) | 94 (TuPV 260, GU214706) |
|  | NS1 | 2085 | 695 | NS1 | 88 (TuPV JO11, KM598421) | 94 (TuPV 260, GU214706) |
|  | NP1 | 305 | 101 | NP1 | 74 (TuPV JO11, KM598421) | 99 (ChPV IPV, KU569162) |
|  | VP1 | 2028 | 676 | VP1 | 73 (TuPV 1090, KM598420) | 94 (TuPV 260, GU214706) |
|  | VP2 | 1611 | 537 | VP2 | 72 (TuPV 1090, KM598420) | 94 (TuPV 260, GU214706) |
| GX-CH-PV-14 | Full genome | 4615 | — | — | 81 (TuPV JO11, KM598421) | 95 (TuPV 260, GU214706) |
|  | NS1 | 2085 | 695 | NS1 | 88 (TuPV JO11, KM598421) | 95 (ParvoD62/2013, KM254172) |
|  | NP1 | 305 | 101 | NP1 | 74 (TuPV JO11, KM598421) | 99 (ChPV IPV, KU569162) |
|  | VP1 | 2028 | 676 | VP1 | 73 (TuPV 1090, KM598420) | 94 (TuPV 260, GU214706) |
|  | VP2 | 1611 | 537 | VP2 | 72 (TuPV 1090, KM598420) | 94 (TuPV 260, GU214706) |
| GX-CH-PV-15 | Full genome | 4615 | — | — | 88 (TuPV JO11, KM598421) | 96 (ChPV IPV, KU569162) |
|  | NS1 | 2085 | 695 | NS1 | 88 (TuPV JO11, KM598421) | 97 (ChPV IPV, KU569162) |
|  | NP1 | 305 | 101 | NP1 | 74 (TuPV JO11, KM598421) | 99 (ChPV IPV, KU569162) |
|  | VP1 | 2028 | 676 | VP1 | 73 (TuPV 1090, KM598420) | 95 (ChPV IPV, KU569162) |
|  | VP2 | 1611 | 537 | VP2 | 73 (TuPV 1090, KM598420) | 94 (ChPV IPV, KU569162) |
| GX-CH-PV-16 | Full genome | 4615 | — | — | 86 (ParvoD11/2007, KM254173) | 90 (ChPV IPV, KU569162) |
|  | NS1 | 2085 | 695 | NS1 | 88 (TuPV JO11, KM598421) | 97 (ChPV 367, KM598414) |
|  | NP1 | 305 | 101 | NP1 | 74 (TuPV JO11, KM598421) | 99 (ChPV IPV, KU569162) |
|  | VP1 | 2028 | 676 | VP1 | 79 (ChPV ABU-P1, GU214704) | 92 (TuPV JO11, KM598421) |
|  | VP2 | 1611 | 537 | VP2 | 74 (ChPV ABU-P1, GU214704) | 96 (TuPV JO11, KM598421) |
| GX-CH-PV-17 | Full genome | 4615 | — | — | 81 (TuPV JO11, KM598421) | 96 (ChPV IPV, KU569162) |
|  | NS1 | 2085 | 695 | NS1 | 88 (TuPV JO11, KM598421) | 97 (ChPV 367, KM598414) |
|  | NP1 | 305 | 101 | NP1 | 75 (TuPV JO11, KM598421) | 99 (ChPV IPV, KU569162) |
|  | VP1 | 2028 | 676 | VP1 | 73 (TuPV 1090, KM598420) | 95 (ChPV IPV, KU569162) |
|  | VP2 | 1611 | 537 | VP2 | 72 (TuPV 1090, KM598420) | 94 (ChPV IPV, KU569162) |
| GX-CH-PV-18 | Full genome | 4614 | — | — | 81 (TuPV JO11, KM598421) | 95 (ChPV IPV, KU569162) |
|  | NS1 | 2085 | 695 | NS1 | 88 (TuPV JO11, KM598421) | 95 (ParvoD62/2013, KM254172) |
|  | NP1 | 305 | 101 | NP1 | 74 (TuPV JO11, KM598421) | 99 (ChPV IPV, KU569162) |
|  | VP1 | 2028 | 676 | VP1 | 74 (TuPV 1090, KM598420) | 95 (ChPV IPV, KU569162) |
|  | VP2 | 1611 | 537 | VP2 | 73 (TuPV 1090, KM598420) | 94 (ChPV IPV, KU569162) |
| GX-CH-PV-19 | Full genome | 4615 | — | — | 88 (TuPV 1085, KM598419) | 98 (GX-CH-PV-16, KX133423) |
|  | NS1 | 2085 | 695 | NS1 | 88 (GX-TU-PV-2, KX084397) | 95 (GX-CH-PV-16, KX133423) |
|  | NP1 | 305 | 101 | NP1 | 90 (ChPV ABU-P1, GU214704) | 100 (GX-CH-PV-14, KX133421) |
|  | VP1 | 2028 | 676 | VP1 | 78 (GX-CH-PV-7, KU523900) | 99 (GX-CH-PV-16, KX133423) |
|  | VP2 | 1611 | 537 | VP2 | 74 (GX-CH-PV-7, KU523900) | 99 (GX-CH-PV-16, KX133423) |
| GX-CH-PV-20 | Full genome | 4615 | — | — | 81 (TuPV JO11, KM598421) | 99 (GX-CH-PV-17, KX133424) |
|  | NS1 | 2085 | 695 | NS1 | 88 (TuPV JO11, KM598421) | 99 (GX-CH-PV-16, KX133423) |
|  | NP1 | 305 | 101 | NP1 | 90 (ChPV ABU-P1, GU214704) | 100 (GX-CH-PV-14, KX133421) |
|  | VP1 | 2028 | 676 | VP1 | 75 (GX-TU-PV-1, KX084396) | 99 (GX-CH-PV-15, KX133422) |
|  | VP2 | 1611 | 537 | VP2 | 75 (GX-CH-PV-8, KX133415) | 100 (GX-CH-PV-15, KX133422) |
| GX-CH-PV-21 | Full genome | 4615 | — | — | 81 (TuPV JO11, KM598421) | 98 (GX-CH-PV-17, KX133424) |
|  | NS1 | 2085 | 695 | NS1 | 88 (TuPV JO11, KM598421) | 100 (GX-CH-PV-16, KX133423) |
|  | NP1 | 305 | 101 | NP1 | 90 (ChPV ABU-P1, GU214704) | 100 (GX-CH-PV-14, KX133421) |
|  | VP1 | 2028 | 676 | VP1 | 75 (GX-TU-PV-1, KX084396) | 99 (GX-CH-PV-15, KX133422) |
|  | VP2 | 1611 | 537 | VP2 | 75 (GX-CH-PV-8, KX133415) | 100 (GX-CH-PV-15, KX133422) |
| GX-CH-PV-22 | Full genome | 4615 | — | — | 81 (TuPV JO11, KM598421) | 95 (GX-CH-PV-14, KX133421) |
|  | NS1 | 2085 | 695 | NS1 | 89 (TuPV JO11, KM598421) | 98 (GX-CH-PV-17, KX133424) |
|  | NP1 | 305 | 101 | NP1 | 90 (ChPV ABU-P1, GU214704) | 100 (GX-CH-PV-14, KX133421) |
|  | VP1 | 2028 | 676 | VP1 | 75 (GX-TU-PV-2, KX084397) | 96 (GX-CH-PV-14, KX133421) |
|  | VP2 | 1611 | 537 | VP2 | 75 (GX-TU-PV-2, KX084397) | 95 (GX-CH-PV-14, KX133421) |
| GX-CH-PV-23 | Full genome | 4615 | — | — | 81 (TuPV JO11, KM598421) | 95 (ChPV IPV, KU569162) |
|  | NS1 | 2085 | 695 | NS1 | 88 (TuPV JO11, KM598421) | 95 (GX-CH-PV-7, KU523900) |
|  | NP1 | 305 | 101 | NP1 | 90 (ChPV ABU-P1, GU214704) | 100 (GX-CH-PV-14, KX133421) |
|  | VP1 | 2028 | 676 | VP1 | 75 (GX-TU-PV-3, KX084398) | 95 (GX-CH-PV-7, KU523900) |
|  | VP2 | 1611 | 537 | VP2 | 74 (GX-CH-PV-8, KX133415) | 95 (GX-CH-PV-7, KU523900) |
| GX-CH-PV-24 | Full genome | 4615 | — | — | 85 (ParvoD11/2007, KM254173) | 94 (GX-CH-PV-11, KX133418) |
|  | NS1 | 2085 | 695 | NS1 | 88 (ParvoD11/2007, KM254173) | 96 (GX-CH-PV-9, KX133416) |
|  | NP1 | 305 | 101 | NP1 | 90 (ChPV ABU-P1, GU214704) | 100 (GX-CH-PV-14, KX133421) |
|  | VP1 | 2028 | 676 | VP1 | 77 (GX-CH-PV-4, KX133401) | 91 (GX-CH-PV-16, KX133423) |
|  | VP2 | 1611 | 537 | VP2 | 73 (GX-CH-PV-14, KX133421) | 91 (TuPV 1030, KM598418) |
| GX-CH-PV-25 | Full genome | 4615 | — | — | 81 (TuPV JO11, KM598421) | 96 (GX-CH-PV-14, KX133421) |
|  | NS1 | 2085 | 695 | NS1 | 88 (TuPV JO11, KM598421) | 99 (GX-CH-PV-16, KX133423) |
|  | NP1 | 305 | 101 | NP1 | 90 (ChPV ABU-P1, GU214704) | 100 (GX-CH-PV-14, KX133421) |
|  | VP1 | 2028 | 676 | VP1 | 75 (TuPV 1090, KM598420) | 97 (GX-CH-PV-14, KX133421) |
|  | VP2 | 1611 | 537 | VP2 | 74 (TuPV 1090, KM598420) | 96 (GX-CH-PV-14, KX133421) |
| GX-CH-PV-26 | Full genome | 4615 | — | — | 81 (TuPV JO11, KM598421) | 96 (GX-CH-PV-17, KX133424) |
|  | NS1 | 2085 | 695 | NS1 | 89 (TuPV JO11, KM598421) | 98 (GX-CH-PV-17, KX133424) |
|  | NP1 | 305 | 101 | NP1 | 90 (ChPV ABU-P1, GU214704) | 100 (GX-CH-PV-14, KX133421) |
|  | VP1 | 2028 | 676 | VP1 | 75 (TuPV JO11, KM598421) | 95 (GX-CH-PV-7, KU523900) |
|  | VP2 | 1611 | 537 | VP2 | 75 (GX-CH-PV-8, KX133415) | 95 (GX-CH-PV-7, KU523900) |
| GX-CH-PV-27 | Full genome | 4615 | — | — | 81 (TuPV JO11, KM598421) | 98 (GX-CH-PV-14, KX133421) |
|  | NS1 | 2085 | 695 | NS1 | 88 (TuPV JO11, KM598421) | 99 (GX-CH-PV-14, KX133421) |
|  | NP1 | 305 | 101 | NP1 | 90 (ChPV ABU-P1, GU214704) | 100 (GX-CH-PV-14, KX133421) |
|  | VP1 | 2028 | 676 | VP1 | 75 (TuPV 1090, KM598420) | 97 (GX-CH-PV-14, KX133421) |
|  | VP2 | 1611 | 537 | VP2 | 74 (TuPV JO11, KM598421) | 96 (GX-CH-PV-14, KX133421) |
| GX-CH-PV-28 | Full genome | 4615 | — | — | 83 (TuPV JO11, KM598421) | 95 (GX-CH-PV-17, KX133424) |
|  | NS1 | 2085 | 695 | NS1 | 89 (TuPV JO11, KM598421) | 99 (GX-CH-PV-16, KX133423) |
|  | NP1 | 305 | 101 | NP1 | 90 (ChPV ABU-P1, GU214704) | 100 (GX-CH-PV-14, KX133421) |
|  | VP1 | 2028 | 676 | VP1 | 79 (TuPV 1090, KM598420) | 92 (GX-CH-PV-18, KX133425) |
|  | VP2 | 1611 | 537 | VP2 | 80 (TuPV 1090, KM598420) | 90 (ChPV 841, KM598417) |
| GX-CH-PV-29 | Full genome | 4615 | — | — | 81 (TuPV JO11, KM598421) | 96 (GX-CH-PV-14, KX133421) |
|  | NS1 | 2085 | 695 | NS1 | 88 (TuPV JO11, KM598421) | 99 (GX-CH-PV-7, KU523900) |
|  | NP1 | 305 | 101 | NP1 | 91 (ChPV ABU-P1, GU214704) | 99(GX-CH-PV-14, KX133421) |
|  | VP1 | 2028 | 676 | VP1 | 75 (TuPV 1090, KM598420) | 96 (GX-CH-PV-14, KX133421) |
|  | VP2 | 1611 | 537 | VP2 | 74 (TuPV 1090, KM598420) | 96 (GX-CH-PV-4, KX084401.1) |
| GX-CH-PV-30 | Full genome | 4615 | — | — | 81 (TuPV JO11, KM598421) | 96 (GX-CH-PV-14, KX133421) |
|  | NS1 | 2085 | 695 | NS1 | 88 (TuPV JO11, KM598421) | 98 (GX-CH-PV-7, KU523900) |
|  | NP1 | 305 | 101 | NP1 | 90 (ChPV ABU-P1, GU214704) | 100(GX-CH-PV-14, KX133421) |
|  | VP1 | 2028 | 676 | VP1 | 75 (TuPV 1090, KM598420) | 96 (GX-CH-PV-14, KX133421) |
|  | VP2 | 1611 | 537 | VP2 | 74 (TuPV 1090, KM598420) | 96 (GX-CH-PV-4, KX084401.1) |
| GX-Tu-PV-1 | Full genome | 4642 | — | — | 80 (ParvoD11/2007, KM254173) | 98 (TuPV 1030, KM598418) |
|  | NS1 | 2088 | 696 | NS1 | 85 (ParvoD11/2007, KM254173) | 98 (TuPV 1090, KM598420) |
|  | NP1 | 311 | 103 | NP1 | 76 (ChPV ABU-P1, GU214704) | 98 (ChPV 841, KM598417) |
|  | VP1 | 2034 | 678 | VP1 | 74 (ChPV 367, KM598414) | 98 (TuPV 1030, KM598418) |
|  | VP2 | 1611 | 537 | VP2 | 73 (ChPV 367, KM598414) | 98 (TuPV 1030, KM598418) |
| GX-Tu-PV-2 | Full genome | 4642 | — | — | 80 (ParvoD11/2007, KM254173) | 94 (TuPV 1030, KM598418) |
|  | NS1 | 2088 | 696 | NS1 | 86 (ParvoD11/2007, KM254173) | 92 (ChPV 841, KM598417) |
|  | NP1 | 311 | 103 | NP1 | 76 (ChPV ABU-P1, GU214704) | 98 (ChPV 841, KM598417) |
|  | VP1 | 2034 | 678 | VP1 | 73 (ChPV 367, KM598414) | 98 (TuPV JO11, KM598421) |
|  | VP2 | 1611 | 537 | VP2 | 72 (ChPV 367, KM598414) | 98 (TuPV JO11, KM598421) |
| GX-Tu-PV-3 | Full genome | 4642 | — | — | 80 (ParvoD11/2007, KM254173) | 98 (TuPV 1030, KM598418) |
|  | NS1 | 2088 | 696 | NS1 | 85 (ParvoD11/2007, KM254173) | 98 (TuPV 1090, KM598420) |
|  | NP1 | 311 | 103 | NP1 | 76 (ChPV ABU-P1, GU214704) | 98 (ChPV 841, KM598417) |
|  | VP1 | 2034 | 678 | VP1 | 73 (ChPV 367, KM598414) | 98 (TuPV JO11, KM598421) |
|  | VP2 | 1611 | 537 | VP2 | 72 (ChPV 367, KM598414) | 98 (TuPV JO11, KM598421) |
| GX-CH-PV-31 | Full genome | 4615 | — | — | 72 (TuPV JO11, KM598421) | 100 (GX-CH-PV-23, MG602513) |
|  | NS1 | 2085 | 695 | NS1 | 88 (TuPV JO11, KM598421) | 99 (GX-CH-PV-23, MG602513) |
|  | NP1 | 305 | 101 | NP1 | 74 (TuPV JO11, KM598421) | 99 (ChPV IPV, KU569162) |
|  | VP1 | 2028 | 676 | VP1 | 73(TuPV 1090, KM598420) | 100 (GX-CH-PV-23, MG602513) |
|  | VP2 | 1611 | 537 | VP2 | 72(TuPV 1090, KM598420) | 100 (GX-CH-PV-23, MG602513) |
| GX-CH-PV-32 | Full genome | 4642 | — | — | 73 (GX-CH-PV-4, KX133401) | 97 (GX-CH-PV-1, KX084399) |
|  | NS1 | 2088 | 696 | NS1 | 85 (ParvoD11/2007, KM254173) | 99 (GX-CH-PV-33, OQ437201) |
|  | NP1 | 311 | 103 | NP1 | 74 (TuPV JO11, KM598421) | 99 (ChPV IPV, KU569162) |
|  | VP1 | 2034 | 678 | VP1 | 73 (GX-CH-PV-4, KX133401) | 97 (GX-CH-PV-1, KX084399) |
|  | VP2 | 1611 | 537 | VP2 | 71 (GX-CH-PV-22, MG602512) | 97 (GX-CH-PV-1, KX084399) |
| GX-CH-PV-33 | Full genome | 4642 | — | — | 73 (GX-CH-PV-22, MG602512) | 97 (GX-CH-PV-32, OQ437200) |
|  | NS1 | 2088 | 696 | NS1 | 85 (ParvoD11/2007, KM254173) | 99 (GX-CH-PV-32,OQ437200) |
|  | NP1 | 311 | 103 | NP1 | 74 (TuPV JO11, KM598421) | 99 (ChPV IPV, KU569162) |
|  | VP1 | 2034 | 678 | VP1 | 73 (GX-CH-PV-22, MG602512) | 97 (GX-CH-PV-32, OQ437200) |
|  | VP2 | 1611 | 537 | VP2 | 71 (GX-CH-PV-22, MG602512) | 96 (GX-CH-PV-32, OQ437200) |

**Table S2 Information of 35 Guangxi ChPV/TuPV and 17 reference strains from other countries**

| **Serial NO.** | **ChPV/TuPV Isolate field Strain ID** | **Origin** | **Isolated date** | **GenBank Accession NO.** | **(G+C)%** |
| --- | --- | --- | --- | --- | --- |
| 1 | GX-CH-PV-1 | China | 2014 | KX084399 | 42.87 |
| 2 | GX-CH-PV-2 | China | 2014 | KX084400 | 42.51 |
| 3 | GX-CH-PV-4 | China | 2014 | KX084401 | 43.08 |
| 4 | GX-CH-PV-5 | China | 2014 | KX133426 | 42.21 |
| 5 | GX-CH-PV-6 | China | 2014 | KX133427 | 42.34 |
| 6 | GX-CH-PV-7 | China | 2015 | KU523900 | 43.34 |
| 7 | GX-CH-PV-8 | China | 2015 | KX133415 | 43.01 |
| 8 | GX-CH-PV-9 | China | 2015 | KX133416 | 42.36 |
| 9 | GX-CH-PV-10 | China | 2015 | KX133417 | 42.98 |
| 10 | GX-CH-PV-11 | China | 2015 | KX133418 | 42.79 |
| 11 | GX-CH-PV-12 | China | 2015 | KX133419 | 42.87 |
| 12 | GX-CH-PV-13 | China | 2015 | KX133420 | 43.13 |
| 13 | GX-CH-PV-14 | China | 2015 | KX133421 | 43.12 |
| 14 | GX-CH-PV-15 | China | 2015 | KX133422 | 42.99 |
| 15 | GX-CH-PV-16 | China | 2015 | KX133423 | 42.69 |
| 16 | GX-CH-PV-17 | China | 2015 | KX133424 | 43.06 |
| 17 | GX-CH-PV-18 | China | 2015 | KX133425 | 43.06 |
| 18 | GX-CH-PV-19 | China | 2016 | MG602509 | 43.14 |
| 19 | GX-CH-PV-20 | China | 2016 | MG602510 | 42.99 |
| 20 | GX-CH-PV-21 | China | 2016 | MG602511 | 42.88 |
| 21 | GX-CH-PV-22 | China | 2016 | MG602512 | 42.67 |
| 22 | GX-CH-PV-23 | China | 2017 | MG602513 | 42.82 |
| 23 | GX-CH-PV-24 | China | 2017 | MG602514 | 42.71 |
| 24 | GX-CH-PV-25 | China | 2017 | MG602515 | 42.99 |
| 25 | GX-CH-PV-26 | China | 2017 | MG602516 | 43.06 |
| 26 | GX-CH-PV-27 | China | 2017 | MG602517 | 42.93 |
| 27 | GX-CH-PV-28 | China | 2017 | MG602518 | 43.06 |
| 28 | GX-CH-PV-29 | China | 2017 | MG602519 | 42.84 |
| 29 | GX-CH-PV-30 | China | 2017 | MG602520 | 42.99 |
| 30 | GX-Tu-PV-1 | China | 2015 | KX084396 | 42.87 |
| 31 | GX-Tu-PV-2 | China | 2015 | KX084397 | 42.80 |
| 32 | GX-Tu-PV-3 | China | 2015 | KX084398 | 43.15 |
| 33 | GX-CH-PV-31 | China | 2021 | OQ437199 | 42.90 |
| 34 | GX-CH-PV-32 | China | 2022 | OQ437200 | 43.21 |
| 35 | GX-CH-PV-33 | China | 2022 | OQ437201 | 43.26 |
| 36 | ChPV IPV | Brazil | 2016 | KU569162 | — |
| 37 | ChPV ADL120019 | South Korea | 2015 | KJ486489 | — |
| 38 | ChPV ADL120035 | South Korea | 2015 | KJ486490 | — |
| 39 | ChPV ADL120686 | South Korea | 2015 | KJ486491 | — |
| 40 | ChPV ParvoD26 | South Korea | 2015 | KM254172 | — |
| 41 | ChPV ParvoD11 | South Korea | 2015 | KM254173 | — |
| 42 | ChPV 367 | USA | 2015 | KM598414 | — |
| 43 | ChPV 736 | USA | 2015 | KM598415 | — |
| 44 | ChPV 798 | USA | 2015 | KM598416 | — |
| 45 | ChPV 841 | USA | 2015 | KM598417 | — |
| 46 | ChPV ABU-P1 | Hungary | 2010 | GU214704 | — |
| 47 | TuPV 260 | USA | 2010 | GU214706 | — |
| 48 | TuPV 1078 | USA | 2010 | GU214705 | — |
| 49 | TuPV 1030 | USA | 2015 | KM598418 | — |
| 50 | TuPV 1085 | USA | 2015 | KM598419 | — |
| 51 | TuPV 1090 | USA | 2015 | KM598420 | — |
| 52 | TuPV JO11 | USA | 2015 | KM598421 | — |

**Table S3 Detect primer information**

| **Primer name** | **Genome region** | **Primer sequence (5’→3’)** | **Product size/bp** | **Annealing temperature/°C** |
| --- | --- | --- | --- | --- |
| NS561F | NS1 | TTCTAATAACGATATCACTCAAGTTTC | 561 | 55 |
| NS561R |  | TTTGCGCTTGCGGTGAAGTCTGGCTCG |  |  |
| VP1 | VP1/VP2 | TGGAATTGTGATACTATATGGG | 373 | 56 |
| VP2 |  | TCYTGATCTGCAAATATTTG |  |  |
| VP3 |  | CATTGTGTCTGTCTWATGCGTGAC | 249 | 64 |
| VP4 |  | GTTTTCTGGATGACTTGCA |  |  |



NS1, nonstructural protein 1; VP1, virion protein 1; VP2, virion protein 2.

**Table S4 Primers used for PCR amplification of the chicken parvovirus genome**

|  | | |
| --- | --- | --- |
| **Primer name** | **Sequence, 5′–3′** | **Position, nt** |
| F1-1 | CTGCTGAGCTGGTAAGATGG | 395-414 |
| R1-2 | TCTTCCCGACTGACTAGATT | 724-743 |
| R1-3 | CCCCCATGATACATTTTGCT | 1751-1770 |
| F2-1 | TTCTAATAACGATATCACTCAAGTTTC | 1841-1867 |
| R1-4 | AACCAGTATAGGTGGGTTCC | 2192-2211 |
| F2-2 | GATCCCCAGCGAATCGAGTGG | 2345-2365 |
| R1-1 | TTTGCGCTTGCGGTGAAGTCTGGCTCG | 2375-2401 |
| F3-1 | CAAGCCGCCATTGTGTTTGT | 3575-3594 |
| R2-1 | GTATTGKGTYTGGTTTTCAG | 3659-3678 |
| R2-2 | AAGTCWAKRTAATTCCATGG | 3694-3713 |
| R3-2 | GTCCCTGTCAAGTCATTAGAG | 3858-3878 |
| F3-2 | CGACGAACAATTCAAAATTA | 4692-4711 |
| R3-1 | TTAATTGGTYYKCGGYRCSCG | 5005-5025 |

Primers F1-1 to R1-1, F2-1 to R2-1 (or R2-2), and F3-1 to R3-1 were used to amplify the first, second, and third fragments of the near complete chicken/turkey parvovirus genome. Primers R1-2, R1-3, R1-4, and R3-2 are also reverse primers.Sequences of primers were designed according to the sequences of three other known ChPV/TuPV strains (GenBank accession no. GU214704, GU214706, NC_024454). Positions of primers located in the complete genome are shown according to the Europe ChPV isolate (Chicken parvovirus ABU-P1).

1. [↑](#footnote-ref-1)
2. [↑](#footnote-ref-2)
3. [↑](#footnote-ref-3)
